# Geometric Deep Learning for Structure-Based Ligand Design

**DOI:** 10.1021/acscentsci.3c00572

**Published:** 2023-11-17

**Authors:** Alexander S. Powers, Helen H. Yu, Patricia Suriana, Rohan V. Koodli, Tianyu Lu, Joseph M. Paggi, Ron O. Dror

**Affiliations:** † Department of Chemistry, Stanford University, Stanford, California 94305, United States; ‡ Department of Computer Science, Stanford University, Stanford, California 94305, United States; § Department of Molecular and Cellular Physiology, Stanford University School of Medicine, Stanford, California 94305, United States; ∥ Department of Structural Biology, Stanford University School of Medicine, Stanford, California 94305, United States; ⊥ Institute for Computational and Mathematical Engineering, Stanford University, Stanford, California 94305, United States; # Department of Bioengineering, Stanford University, Stanford, California 94305, United States; ∇ Biomedical Informatics Program, Stanford University School of Medicine, Stanford, California 94305, United States

## Abstract

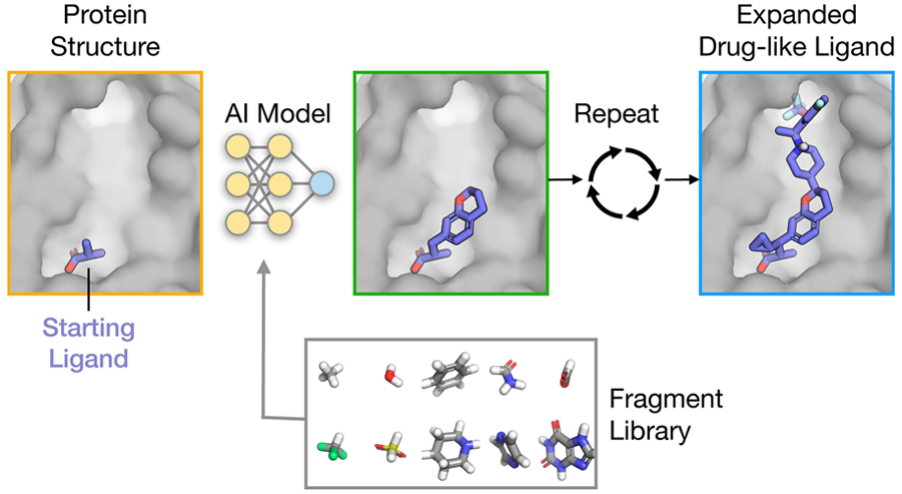

A pervasive challenge in drug design is determining how to expand a ligand—a small molecule that binds to a target biomolecule—in order to improve various properties of the ligand. Adding single chemical groups, known as fragments, is important for lead optimization tasks, and adding multiple fragments is critical for fragment-based drug design. We have developed a comprehensive framework that uses machine learning and three-dimensional protein–ligand structures to address this challenge. Our method, FRAME, iteratively determines where on a ligand to add fragments, selects fragments to add, and predicts the geometry of the added fragments. On a comprehensive benchmark, FRAME consistently improves predicted affinity and selectivity relative to the initial ligand, while generating molecules with more drug-like chemical properties than docking-based methods currently in widespread use. FRAME learns to accurately describe molecular interactions despite being given no prior information on such interactions. The resulting framework for quality molecular hypothesis generation can be easily incorporated into the workflows of medicinal chemists for diverse tasks, including lead optimization, fragment-based drug discovery, and *de novo* drug design.

## Abstract 2

We developed an AI-driven method to expand lead molecules into drug-like ligands with desirable properties, facilitating structure-based drug design.

## Introduction

The drug discovery process is increasingly long and expensive.^1,2^ A major challenge at all stages of this process is choosing new molecules to synthesize and test. By selecting the optimal candidates from the enormous space of possible molecules, one can save time and money on resource-intensive testing and ultimately find more viable therapeutics.^3^

A key part of this design process is adding chemical groups (termed “fragments” in this work) to a starting molecule known to bind to the target in order to tune its properties such as affinity, selectivity, and solubility.^4^ For example, lead optimization generally involves adding one or two fragments at a time to a starting molecule, followed by iterative rounds of testing and further modifications.^5,6^ In other cases, it is useful to add multiple fragments; in fragment-based drug discovery, multiple fragments are often added to a small starting molecule obtained from structural screening methods.^7−9^ Expanding these starting molecules can result in more desirable drug properties, such as higher affinity and specificity for the protein target.^10−12^ However, in practice it is still exceedingly difficult to propose the optimal expansions as the desired properties are hard to predict *a priori* and the space of possible chemical modifications is vast.^13−15^

We have developed a comprehensive framework, Fragment-Based Molecular Expansion (FRAME), that uses machine learning and three-dimensional protein–ligand structures to address this common challenge in drug design. FRAME represents the expansion process as a sequence of steps in 3D space. Given an input structure of a starting molecule bound to a protein pocket, FRAME determines where to attach fragments, selects the fragments to add, and determines the fragments geometry. Rather than hard-coding rules about synthesizability or affinity, we train neural networks to recognize patterns from existing structures of high-affinity, drug-like ligands bound to proteins. With recent innovations in structure determination, these data sets are rapidly increasing in size;^16,17^ FRAME will improve along with new discoveries and approaches reflected in these data sets. Though not a substitute for a trained medicinal chemist, FRAME is highly effective for quick hypothesis generation, suggesting candidates that chemists and biologists can then evaluate and experimentally test.

Our approach differs substantially from existing strategies currently in use, such as virtual screening with physics-inspired scoring functions (ligand docking).^18−20^ First, our approach does not require systematically evaluating every candidate molecule as usually done in virtual screening, enabling FRAME to explore a much larger chemical space.^19^ For example, in five expansion steps, FRAME can express over 300 billion unique molecules, which would typically take weeks to evaluate with docking. Yet, our method can intelligently sample promising regions of this large chemical space in a few minutes. Furthermore, FRAME does not use docking scores or expert-crafted rules typically employed in virtual screening, which may contain biases and pitfalls.^21^ Thus, FRAME can be used to provide an orthogonal source of design hypotheses compared to optimization methods that use docking scores.^22−25^

Likewise, many existing machine learning methods for generating molecules are not directly suitable for structure-based drug design as they do not leverage the 3D structure of a target.^26−29^ A handful of recent machine learning methods do utilize protein pocket structure for molecule generation, but these methods are designed for different tasks than FRAME. For example, some methods generate completely new molecules, not expansions.^30,31^ Others predict a single fragment to add to a preselected location,^32^ whereas FRAME attempts a more complex sequence of actions with no restriction on attachment location or number of added fragments. FRAME overcomes these limitations and provides a flexible framework for expanding molecules applicable to diverse drug design tasks.

## Methods

### Expanding Molecular Structures

**Figure 1:**
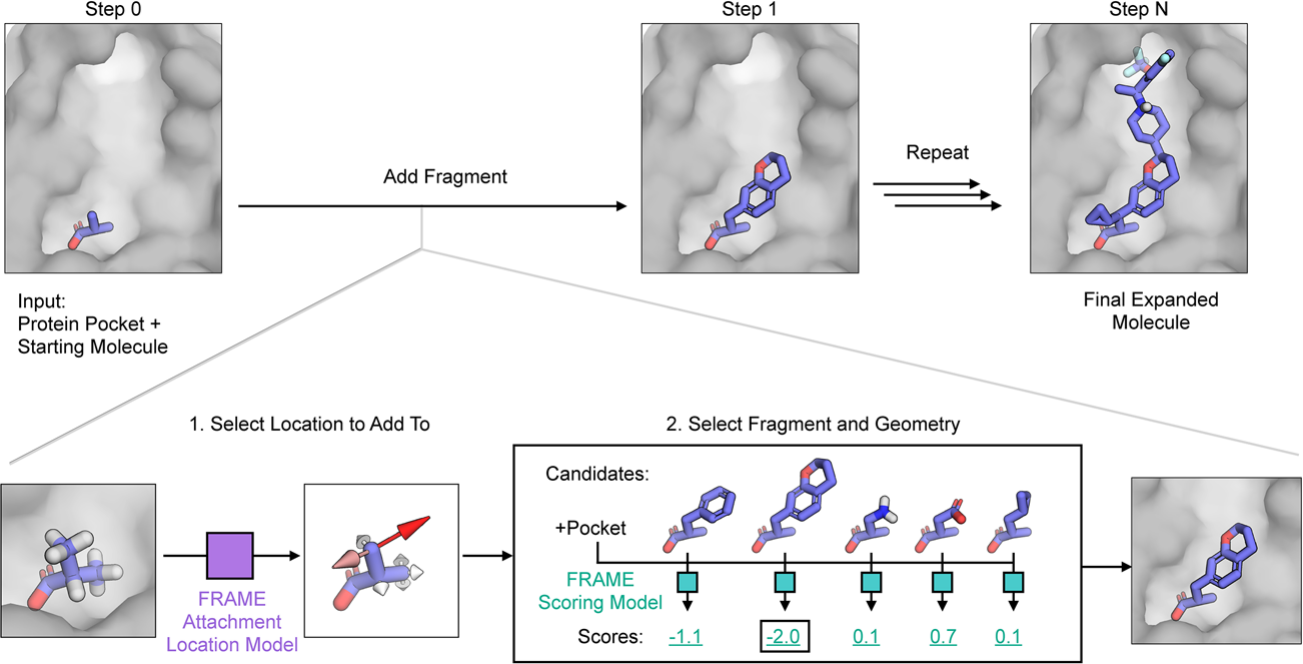
Overview of FRAME: FRAgment-based Molecular Expansion. Generation begins with a starting molecule (purple sticks) placed within a protein pocket (gray surface; step 0). The method sequentially adds fragments to the molecule, connected by single bonds, until the molecule reaches a user-specified goal, such as molecular weight or a predicted stopping point (step N). Each action is broken down into two steps: location selection and fragment selection. First, to select a location to attach a fragment, potential attachment points are assigned a score by the Attachment Location Model (purple), an SE(3)-equivariant neural network. The network is trained to recognize likely attachment points using curated structures of the ligand–protein complexes. After selecting the highest scoring location (red arrow), we generated a set of candidate structures by sampling fragments and geometries (only a small selection of fragments is shown here). These candidate structures are scored by the Fragment Scoring Model (turquoise), which again is an SE(3)-equivariant neural network trained using a data set of known ligand–protein complexes. The best-scoring state is selected, and the process is repeated (step 1).

FRAME uses trained neural networks to select actions that expand a ligand molecule based on the current molecular structure. Initially, the structure consists of the starting molecule (of any size) as well as the protein pocket including the 3D coordinates and element type of each atom (Figure 1). FRAME sequentially adds fragments to the ligand, connected by single bonds. Each action is broken down into two steps: first, selecting a location to attach a fragment and, second, choosing which fragment to add and the attachment geometry. We train two separate SE(3)-equivariant neural networks to make predictions for each step (Figure 1).^33,34^ The fragments are selected from a user-specified library; for benchmarking, we used a library of common fragments (Figure S1). Additional fragments can be easily included in the library, as FRAME is able to evaluate fragments not seen in the training data.

The FRAME neural networks are trained by using a curated set of protein–ligand structures from the Protein Data Bank (PDB), termed *reference ligands*. Each reference ligand structure is broken down to produce a sequence of intermediate molecular structures, termed *reference trajectories* (see Supporting Information). As each ligand in our data set is synthesizable and has relatively high affinity to the corresponding target (median *K*_D_ = 300 nM), its structure and constituent fragments are to some extent optimized and desirable compared to a random molecule. We therefore trained the networks to reconstruct the trajectories of these known active ligands. Although this approach may not necessarily output the single best ligand, the immediate goal is to produce realistic candidates for consideration by medicinal chemists or other algorithms. Thus, the challenge is for the FRAME networks to learn generalizable rules (favorable interactions and synthetic feasibility) that produced these reference ligands rather than simply memorizing them.

To simplify the problem and make it amenable to efficient supervised learning, we break up the trajectories from the reference ligands into individual labeled examples. To predict attachment locations, we consider ligand hydrogen atoms as potential attachment points for the new fragments. Using the reference trajectories, we computed binary labels for each ligand hydrogen atom in the intermediate steps, corresponding to whether the hydrogen would be replaced by a fragment in the final step. We then trained a neural network to predict these labels. The resulting FRAME attachment location model scores each ligand hydrogen atom to determine whether it should be used as an attachment point (Figure 1).

For the fragment selection step, we trained a scoring network that is used to rank the candidate fragments. The resulting FRAME fragment scoring model outputs a numerical score given a structure with the candidate fragment attached (Figure 1). Candidate structures are generated by enumerating fragments from the library, distinct attachment points on each fragment, and fragment dihedral angles to a specified resolution. The training data are derived from the intermediate states of the reference ligand trajectories. The scoring network is trained to assign a favorable (negative) score to the reference intermediate states and an unfavorable (positive) score to the decoy states. The decoy states are created by randomly attaching other fragments or sampling other geometries. We incorporated several fine-tuning strategies; we added more challenging decoys and weighted the examples depending on the types of interactions formed with the protein pocket (see Supporting Information).

The molecular expansion process can output a single ligand by greedily choosing the highest scoring actions at each step. Alternatively, the FRAME model scores can be used to inform more sophisticated search strategies that produce a set of diverse ligands. In this work, we focus on the greedy case and leave other search strategies to future work. The molecule expansion can continue until a user-specified goal is reached such as molecular weight or number of atoms. Alternatively, FRAME can automatically detect an end point when the attachment location model outputs no predicted attachment points.

### Data Sets

For the training and testing data sets, we curated a collection of high-resolution 3D structures containing drug-like, relatively high-affinity ligands. An initial set of ligand–protein complexes were obtained from the PDBBind data set.^35,36^ The data set was filtered to remove common biomolecules (lipids, peptides, carbohydrates, and nucleotides), duplicate ligands, and compounds outside property ranges. This resulted in a data set of 4200 ligands, with drug-likeness scores similar to those of FDA approved drugs (Figure S2). Reference trajectories were created by sequentially removing fragments from each ligand. We then derived two data sets: a data set used to evaluate the location to add fragments and a data set to score candidate fragments. We split both data sets using the same split of protein–ligand pairs into training (70%), validation (15%), and test (15%) sets. To assess the generalizability across diverse proteins, we split these structures such that no protein in one set had more than 30% sequence identity with any protein in the other sets. To improve computational efficiency, we included only amino acid residues in each binding pocket. To prepare the pocket structures, we selected residues in close proximity to the reference ligand. However, we also added noise to this selection process to avoid the possibility of revealing information to the model about the exact positions of the reference ligand atoms (see Supporting Information).

We created a custom fragment library by combining curated fragments relevant to drug discovery with automatically detected fragments from the ligand data set (Figure S1, Supporting Methods). We also accounted for differing protonation and tautomeric states of the fragments. The data set consists of 900 unique fragments; however, the vast majority of these occurred very rarely (Figure S1). To train the model, we used all of the available fragments in the training set. For benchmarking tasks, we selected a subset of the 60 most frequently occurring fragments, which strikes a balance between efficiency and expressivity (Figure S1). As the fragment identities are not explicitly encoded within the scoring models, the fragment library can be adjusted to the specific application.

### Architecture and Training

To predict scores from atomic structures, we used SE(3)-equivariant neural networks, which capture the precise geometry of the ligand relative to the protein pocket.^33,34,37^ These neural networks consist of several layers, with each layer’s outputs serving as the inputs of the next layer. The first layer’s only inputs are the 3D atomic coordinates, chemical element type of each atom, and flags indicating whether an atom belongs to the ligand, protein, or candidate fragment when applicable. We did not use any hand-crafted features or other computed properties. Each layer then computes new features for each atom based on the geometric arrangement of surrounding atoms and the features computed by the previous layer. These SE(3)-equivariant neural networks take into account the geometry of all atoms in the pocket, ligand, and candidate fragment, including hydrogens, allowing the FRAME to implicitly consider factors such as ionizable chemical groups, stereocenters, and molecular strain.

The final layers of the network aggregate information across sets of atoms to produce scores. For scoring fragments, the embedded features of the candidate fragment atoms are aggregated and passed through dense neural network layers to yield the final score. For the attachment location model, the final embedded features of each ligand hydrogen atom are passed independently through dense neural network layers, which produces a list of scores corresponding to each hydrogen atom. These scoring networks are rotationally and translationally invariant—that is, rotation or translation of the input structures does not affect the output scores, improving training efficiency and generalizability.

## Results and Discussion

### FRAME Learns to Rank Individual Fragments in the Context of the Binding Site

Before applying the full capabilities of FRAME to add multiple fragments iteratively, we assessed the model performance on the simpler tasks of selecting attachment points and ranking fragments.

**Figure 2:**
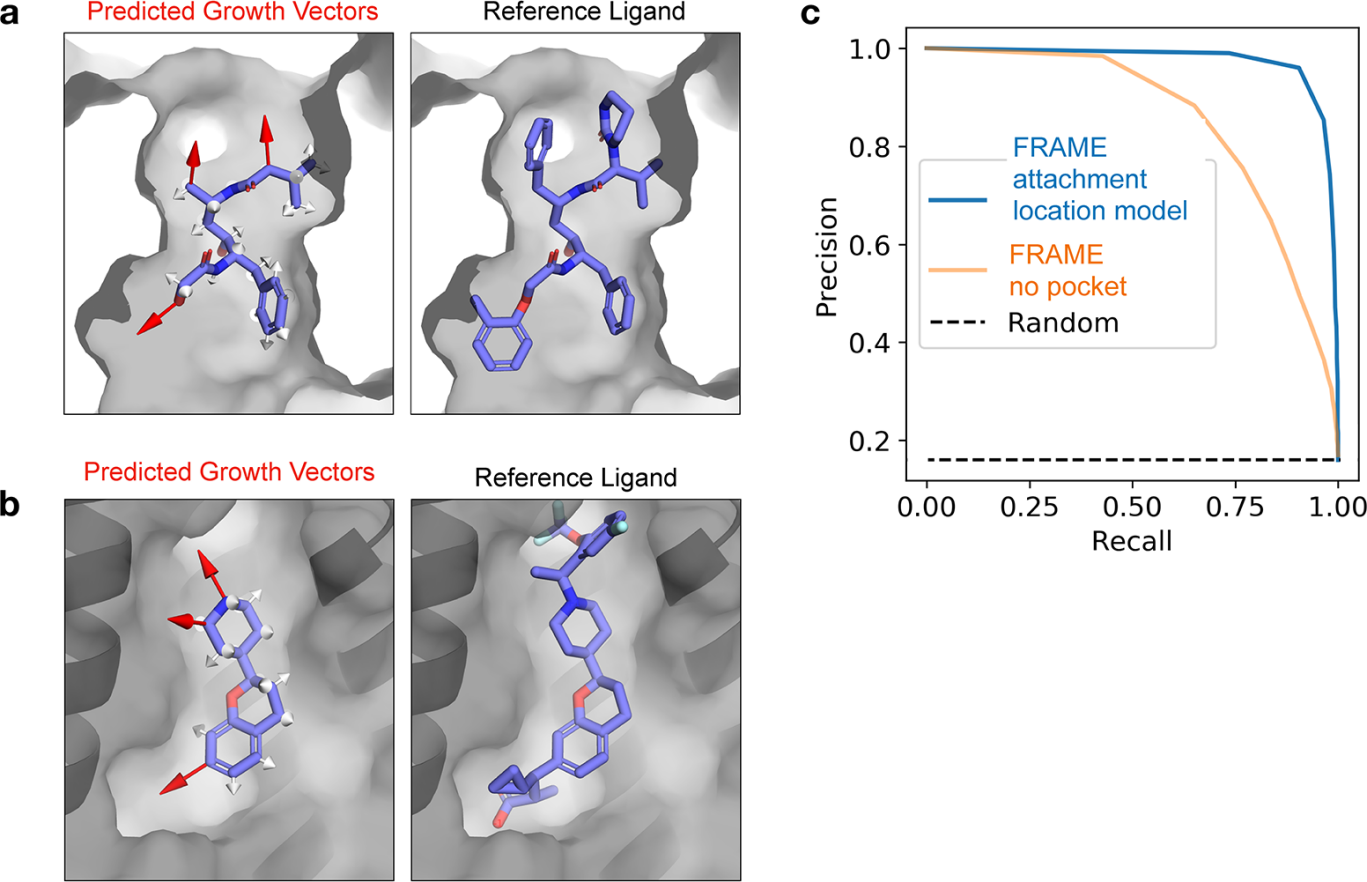
FRAME learns where to attach fragments in order to expand a ligand in the three-dimensional context of the protein pocket. (a, b) FRAME identifies optimal attachment points for fragments (visualized as growth vectors here) by scoring the ligand hydrogen atoms using a trained neural network. In the images, red arrows indicate high-scoring attachment points (score ≥ 0.5), while white arrows indicate low-scoring ones. Comparison with the complete reference ligands (right images) shows that FRAME often selects the actual attachment points utilized in these ligands. Panel a shows HIV1 protease (PDB 2Q5K), and panel b shows an allosteric pocket of FFAR1 (PDB 5TZY). (c) To evaluate performance, we plotted a precision–recall curve for FRAME’s ability to correctly identify the attachment points used in reference ligands given intermediate states. Precision is the fraction of predicted positive data points that are true positives (actual attachment points in reference ligands), while recall is the fraction of true positive data points correctly predicted as positive. Curves are drawn by calculating the precision and recall at varying score thresholds. FRAME (solid blue line) achieves a performance well above a random baseline (dashed line). To test the relevance of the protein pocket information, we trained an additional model (“FRAME no pocket”, solid orange line) that excluded the pocket and observed that the performance was reduced. The data are derived from 700 examples from the test set.

First, we found that the FRAME attachment location model selects viable locations to attach fragments. From a visual inspection, the model frequently identified unobstructed attachment locations that pointed toward unfilled areas within the protein pocket (Figure 2a,b). Given intermediate states from the test set reference ligand trajectories, FRAME often selects the actual attachment points utilized in the reference ligands. Quantitatively, 95% of the points selected by the model are attachment points in reference ligands (precision), and 92% of the reference attachment points are selected by the model (recall). The FRAME model far outperforms a random baseline (Figure 2c), and overall, the model generalizes well for this task.

FRAME considers the atoms of both the partial ligand and protein pocket when predicting attachment locations. Information from the ligand atoms may allow FRAME to evaluate chemical synthesizability, while the pocket informs the steric effects and interactions. To test the relative importance of pocket information, we trained the network only on the partial ligand atoms (no protein atoms). The performance was degraded (recall 70%, precision 80%) though still better than the random baseline, indicating the importance of both ligand and pocket information (Figure 2c).

**Figure 3:**
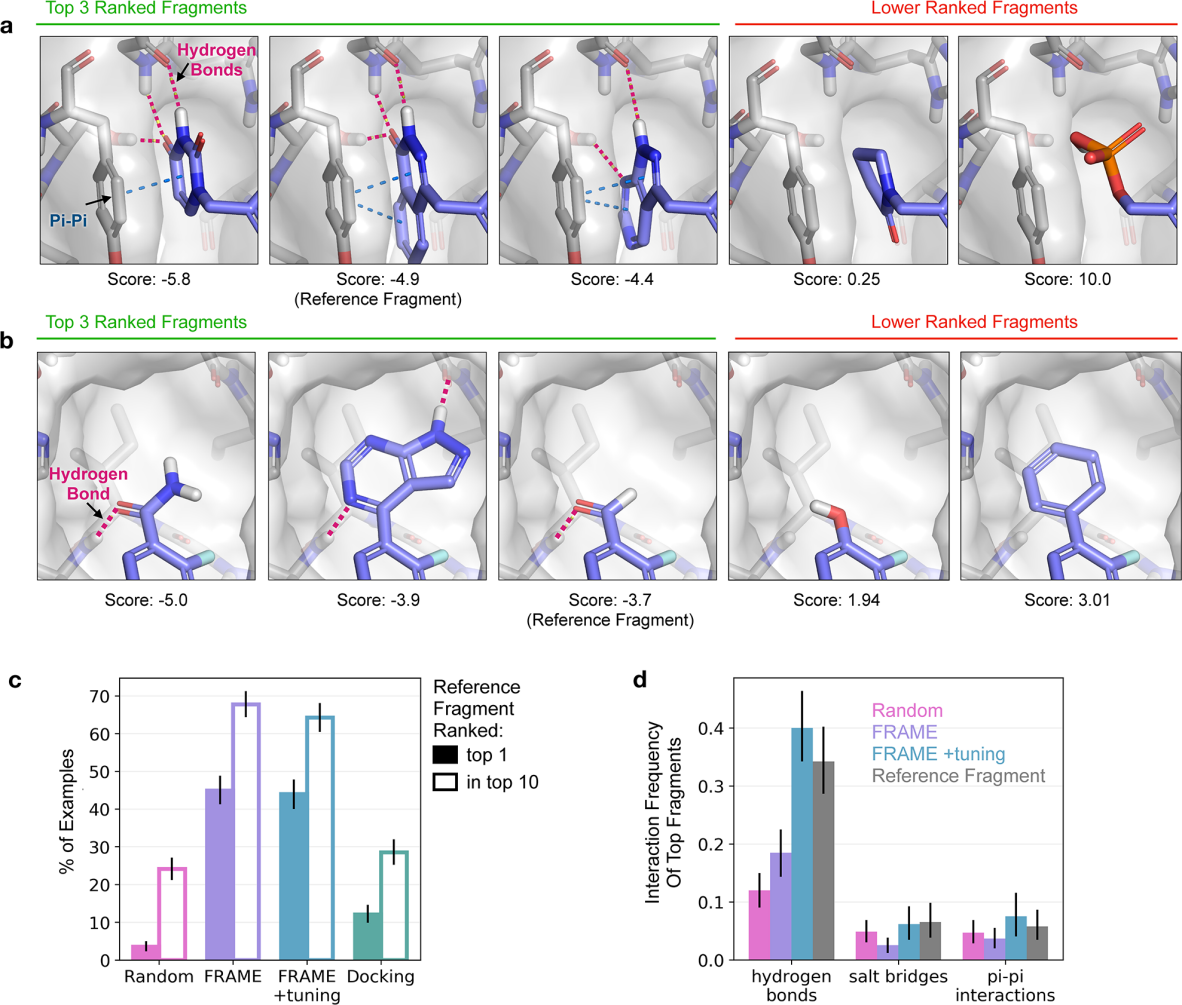
FRAME scoring model selects fragments that form key interactions with the pocket, as desired in ligand optimization. (a, b) FRAME ranks fragments by scoring each candidate structure with a learned model. Examples show the top 3 ranked fragments (with best scoring geometries) at left, with selected lower ranked fragments at right. The reference fragment is that found in the reference ligand from the data set. Hydrogen bonds are indicated by dotted red lines, and π–π interactions by dotted blue lines. These two examples are from PAR polymerase (PDB 3C49). (c) We evaluated the fragment-scoring model by measuring how often it ranked the reference fragment first (filled bars) or within the top 10 fragments (outlined bars). We compared to a random baseline (Random), a version of the model fine-tuned with weighted examples (FRAME + tuning), and docking scores (Docking). The analysis used 100 ligand–protein complexes from the test set, and error bars indicate the 95% confidence interval obtained from bootstrapping. (d) We also evaluated the frequency of interactions made by the top-ranked fragments. The frequency value corresponds to the average number of interactions formed by the added fragment. Bar colors correspond to the methods described in (c) along with the reference fragment (gray). The analysis used 100 ligand–protein complexes from the test set, and error bars indicate the 95% confidence interval obtained from bootstrapping.

Next, we evaluated FRAME’s ability to select single fragments at a given attachment point, which is applicable for ligand optimization tasks where users want a ranked list of candidate fragments to consider. We found that the model often selects fragments that form key interactions, despite the model having no prior knowledge of these interactions or even chemical properties like donors and acceptors (Figure 3a,b). In one case study, FRAME selected ring fragments that formed both π–π interactions and multiple hydrogen bonds—important interactions found in the reference ligand (Figure 3a). Notably, FRAME correctly distinguished these heterocycles from other rings that could not form the same interactions. FRAME was also capable of enriching diverse fragments. In another example, the top three fragments differ in size and chemical properties, but all form the same key hydrogen bond with the pocket (Figure 3b).

FRAME successfully recovers fragments found in reference ligands (termed *reference fragments*) and does so at a rate higher than that of docking scores. As reference fragments are optimized relative to random fragments, the ability to enrich reference fragments is an important quantitative measure of the model’s performance. On test set examples, the fragment-scoring model selects the reference fragment as the top choice 45% of the time and within the top 10 fragments 65% of the time, which is approximately 3 times higher than random choice or using docking scores (Figure 3c).

FRAME selects fragments that form key interactions with the protein pocket, which is important for ligand affinity and specificity. Not all fragments in a ligand are of equal significance; interactions such as salt bridges are rare but often essential for a functional effect.^38^ The fine-tuned version of the FRAME model selected fragments that form specific interactions at a rate similar to that of reference fragments, including salt-bridges and π–π interactions (Figure 3d, Table S1). We also measured the model’s ability to select fragments that specifically recover the interactions of the reference fragments; the fine-tuned version of the model recovers the interactions 78% of the time (Figure S3, Table S2). In contrast, docking scores select fragments that form far more interactions than reference fragments (Table S1 and Table S2). Pending experimental evaluation, it is unclear if these extra interactions are in fact deleterious to binding.

We also explored the robustness of FRAME to perturbations of the starting molecule such as translations and rotations. The fragment ranking was generally robust to perturbations with translation distances less than 0.5 Å and rotations less than 10° (Figure S4). We found one possible strategy to address larger perturbations is to first apply a force-field minimization of ligand coordinates prior to FRAME scoring (Figure S4d).

### FRAME Learns to Adds Multiple Fragments to Produce Drug-Like Ligands

We next applied FRAME to a fragment-based drug discovery scenario in which multiple fragments are attached to expand a small starting molecule. This task requires iterative application of the attachment and fragment selection models, which is a substantially harder problem than scoring single fragments. To evaluate the performance for this task, we applied FRAME to expand 100 molecules for 100 unique test protein pockets, using randomly selected small substructures from the reference ligands as starting molecules. We produced one expanded molecule per pocket. To simplify comparisons, we stopped adding fragments to the ligand once it reached a similar number of heavy atoms to that of the corresponding reference ligand, avoiding significant size differences. For comparison, we also generated ligands using *iterative docking*; a state-of-the-art physics-based scoring function (Glide, see Supporting Information) was used in place of FRAME scoring to select and position a fragment at each step. This approach is widely employed in molecular design software.^22,39,40^

**Figure 4:**
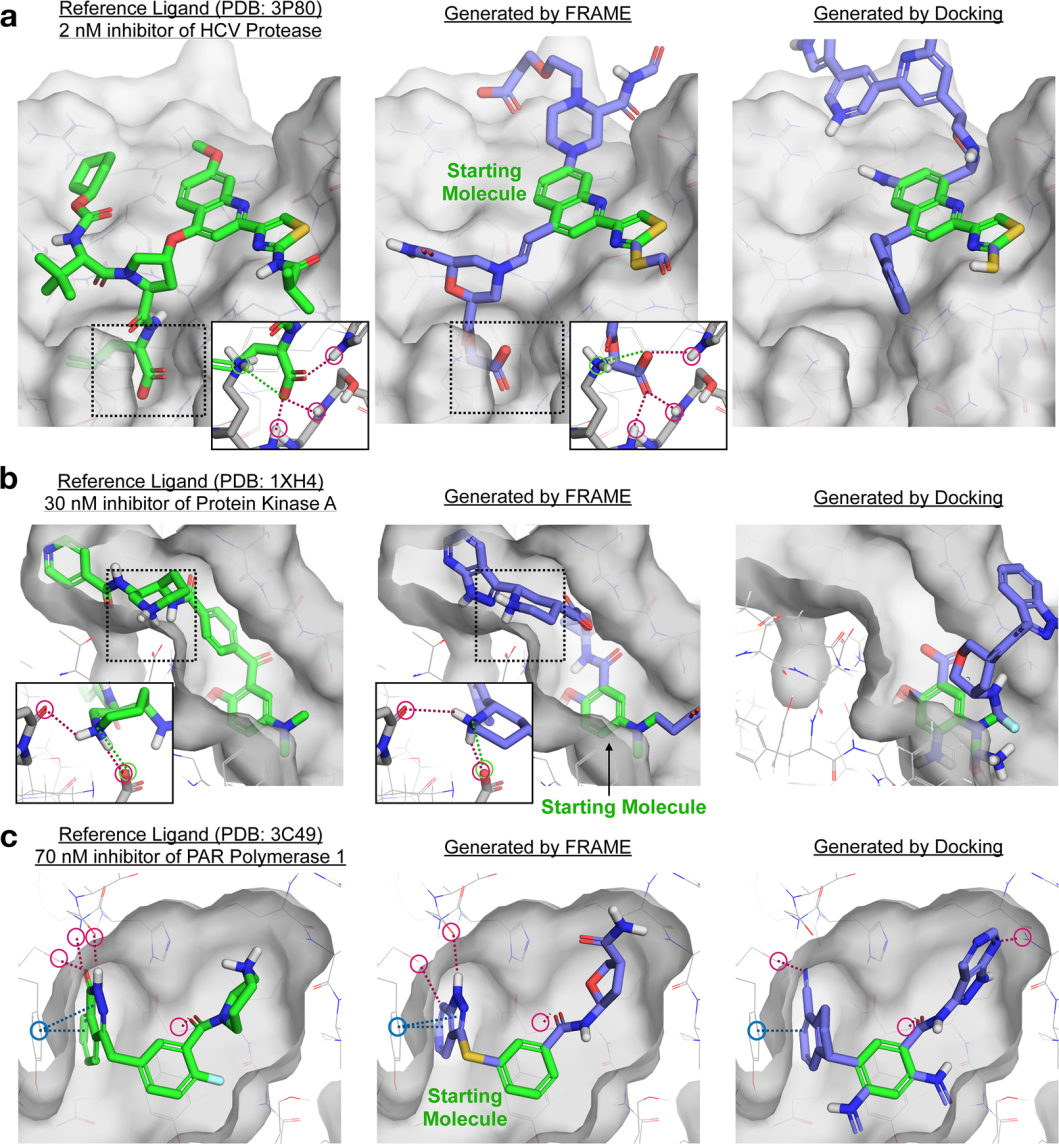
FRAME successfully expands small starting ligands by adding multiple fragments, as desired in fragment-based drug design. Reference ligands (green sticks) and pockets (gray surface) for three example proteins from the test set are shown in the left column. A small starting molecule was randomly selected from the reference ligand to initiate expansion; the starting molecule is shown in green in the middle and right columns. The starting molecules were expanded using FRAME to select attachment points and fragments; the resulting molecules are shown in the middle column (added fragments shown in purple). We compared these to molecules generated using iterative docking (with Glide) to select fragments, right column. A single expanded molecule (the one shown in each image) was generated per pocket for each method. Key interactions are highlighted by circles and dotted lines: hydrogen bonds in red, π–π interactions in blue, and salt bridges in green. (a, b) Detail images show key residues on the protein pocket (gray sticks) and interactions with ligands.

FRAME often generates ligands that form key interactions and fill out the pocket similarly to reference ligands, as demonstrated in several case studies with test set proteins (Figure 4). As a challenging case study, we applied FRAME to design inhibitors of hepatitis C virus protease, which presents a mostly shallow, solvent-exposed binding site (Figure 4a).^41^ The randomly chosen starting molecule is distant from the catalytic site needed for high affinity, requiring several precisely placed fragments to reach it. Promisingly, FRAME was able to expand toward the active site and place a carboxylate fragment in an optimal location to form interactions with the catalytic site residues.^41^ Because FRAME learns to imitate ligand growth trajectories, it learns to expand molecules in beneficial directions even when multiple fragments must be added before the expanded ligand forms energetically favorable interactions. In contrast, the ligand generated with iterative docking failed to enter the catalytic site at all. Standard docking scores evaluate only immediate interactions at each step, so iterative docking does not anticipate advantageous growth directions that require the addition of multiple fragments over several steps in order to form favorable interactions.

In another example, FRAME was applied to design inhibitors of protein kinase A starting from a minimal starting molecule.^42^ Again, FRAME was able to extend the ligand to make critical interactions, this time by adding a protonated piperidine ring (Figure 4b). This mimics the azepane ring in the reference ligand that is known to be essential for binding.^43^ The ligand generated with iterative docking failed to form these interactions. We also investigated a simpler case that requires less expansion steps: inhibitors of poly(ADP-ribose) polymerase of interest for cancer therapy.^44^ FRAME was able to again extend the ligand effectively (Figure 4c); a heterocyclic ring extends into a cleft to make π–π interactions and hydrogen bonds, while an amide links to an aliphatic ring that occupies a shallow pocket. These features are also present in the reference ligand. While iterative docking performs better in this simpler example than those discussed previously, it adds several extraneous fragments that increase the synthetic complexity.

**Figure 5:**
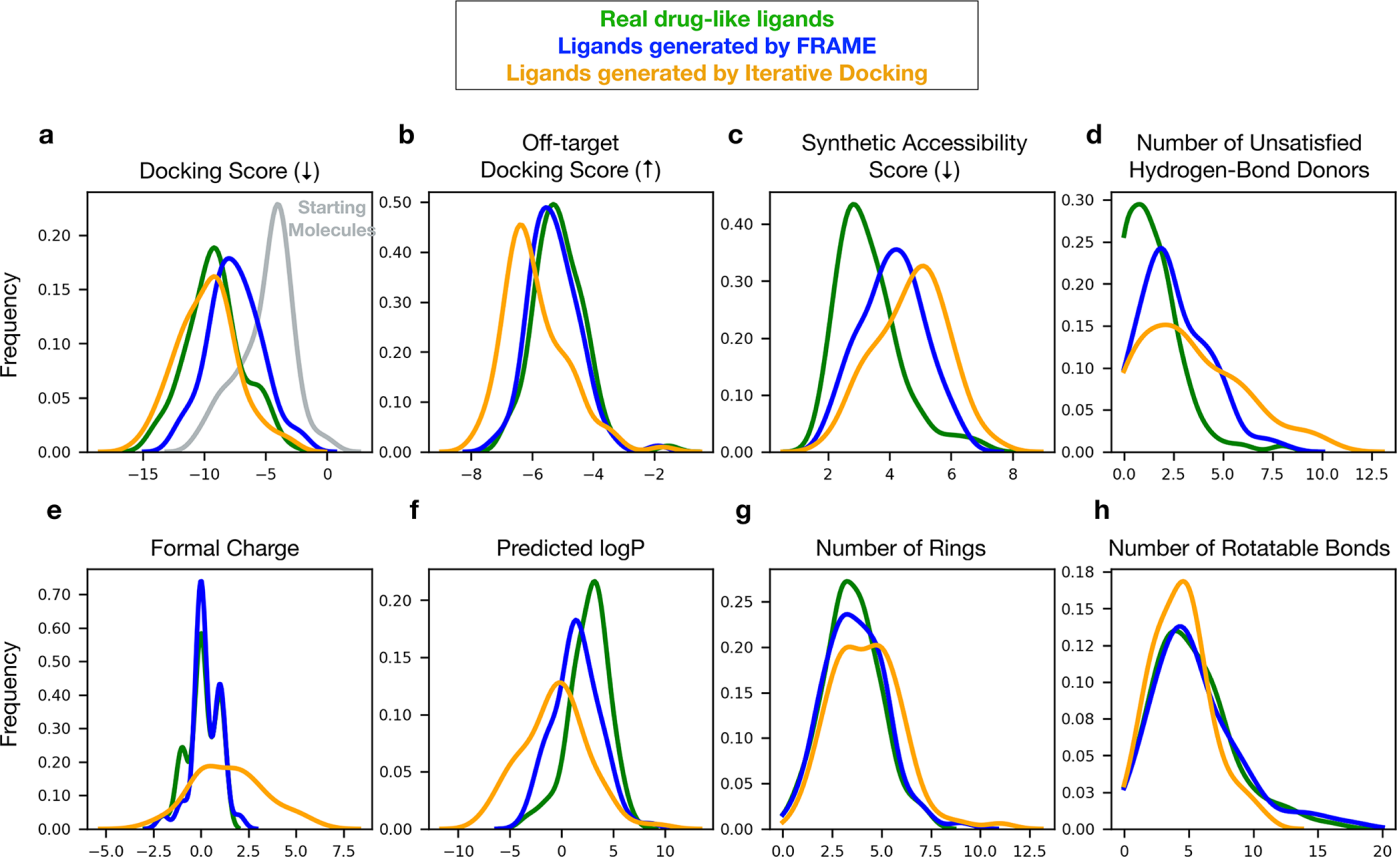
FRAME improves predicted affinity relative to starting molecules while maintaining more drug-like chemical properties compared to other methods. 100 ligands were expanded using FRAME, using 100 unique structures from the test set. A small starting molecule was randomly selected from the reference ligand to initiate expansion, and multiple fragments were added. The property distributions of the expanded ligands were compared to those of ligands expanded by using iterative docking (with physics-based docking software Glide). The property distributions of the reference ligands are also included. The number of heavy atoms of the reference ligand was used to determine when to stop adding fragments for a particular example. Distributions are plotted using kernel density estimations. (a) Docking scores are Glide scores at the target protein obtained after restrained minimization of the ligands (lower is better). Distribution of starting molecules is also shown (gray). (b) Off-target docking scores are the scores of ligands when docked against the other 99 pockets in the test set (higher is better). (c) Synthetic accessibility score, calculated using the standard implementation in RDKit, is a relative measure of the ease of synthesizing the ligand, with lower scores indicating easier synthesis. (d) Number of hydrogen bond donors on the ligand that do not form any hydrogen bond with the protein pocket; generally lower is better to avoid a high desolvation penalty. (e–h) All other properties are standard molecular descriptors computed with RDKit.

We next quantitatively evaluated the properties of ligands generated by FRAME and found that they matched the drug-like reference ligands across many key features. We also compared three alternative methods for generating ligands. First, we employed iterative docking, as discussed above. Second, we used *random expansion*, in which we randomly selected a fragment that did not clash with the pocket or form unstable bonds. Third, we compared to *virtual screening* using a commercial library of over 30 billion ligands (Enamine REAL Space) and state-of-the-art docking software (see Supporting Information). For each set of generated ligands, we measured a panel of 20 properties (Figure 5, Figure S5) including log *P*, synthetic complexity, and docking scores (using the physics-based scoring function Glide). We note that docking scores are used here as an indicator of binding affinity, with the caveat that they provide only a rough estimate of affinity.

The FRAME-expanded ligands improved the predicted binding affinity relative to starting molecules, as estimated by median docking scores after energy minimization (−7.54 vs −4.36 kcal/mol; Figure 5a, Figure S5). We also evaluated specificity by docking the expanded ligands to all other nontarget pockets: FRAME improved the docking scores more for the targets than the nontargets (Figure 5a,b). The median docking scores of the generated molecules were not as low as those of the reference ligands (−9.17 kcal/mol), although they were better than those of ligands from random expansion (−5.45 kcal/mol). FRAME also excelled at producing ligands similar to the reference ligand in chemical features, such as formal charge, number of rings, and number of rotatable bonds (Figure 5e,g,h). Molecules generated by FRAME had a median synthetic complexity score slightly higher than that of reference ligands, likely due to a higher number of stereocenters (Figure 5c, Figure S5).

Molecules produced with iterative docking tended to have favorable final docking scores (Figure 5a), but they were more charged and polar than reference ligands (Figure 5e, f). Iterative docking also produced ligands with the highest median synthetic complexity of the methods assessed (Figure 5c). We note that the favorable docking scores of ligands generated by iterative docking are unsurprising given that this method *explicitly* optimizes for docking scores. This may tend to generate ligands whose binding affinity is overestimated when also evaluated with docking scores.^45^ By contrast, FRAME makes no use of docking scores internally but still manages to improve them. Experimental measurements will be necessary to determine with confidence how affinities of FRAME-generated ligands compare to the affinities of those generated by docking methods.

FRAME also outperformed the virtual screening approach. For 27 of the 100 proteins in the benchmark, the virtual screening approach was unable to generate any ligands because no molecule in the large commercial library contained the starting molecule as a substructure. In the remaining 73 cases, the virtual screening method did generate ligands, but these were overall of lower quality than those produced by FRAME (Figure S5). For example, the median docking score of the molecules selected by virtual screening was −6.8 kcal/mol, worse than the median docking score of the FRAME-generated molecules at −7.5 kcal/mol—despite the fact that the virtual screening method was specifically designed to select ligands with the best docking scores.

As a final comparison of ligands produced by each method, we calculated an overall quality score using the statistical similarity of the property distributions shown in Figure 5 to the reference ligand distributions (Figure S6). In this evaluation, we also included LiGAN, a recent machine learning method that generates complete ligands (not expansions) using density grids and convolutional neural networks (Figure S7). Ligands produced with FRAME had the highest overall quality compared to the other methods evaluated.

To conclude our evaluation, we also confirmed that FRAME’s performance was robust to changes in the input protein pocket structure and in particular that FRAME can be applied effectively to the type of structures available at the beginning of a ligand optimization process. In the preceding benchmark (Figure 5) and in FRAME training data, the protein structures we started with were each determined with a large, high-affinity ligand bound. At the beginning of a ligand optimization process, a structure would often be available only with a small, fragment-like ligand bound. We thus constructed an additional benchmark data set consisting of a pair of structures for each of 100 proteins—one structure determined with a large ligand bound and the other determined with a substantially smaller ligand bound (Figure S8). We found that FRAME’s performance did not depend on the ligand present in the experimentally determined structure, even when that ligand was only a small fragment (Table S3 and Figure S8).

### FRAME Learns to Recognize Molecular Interactions

**Figure 6:**
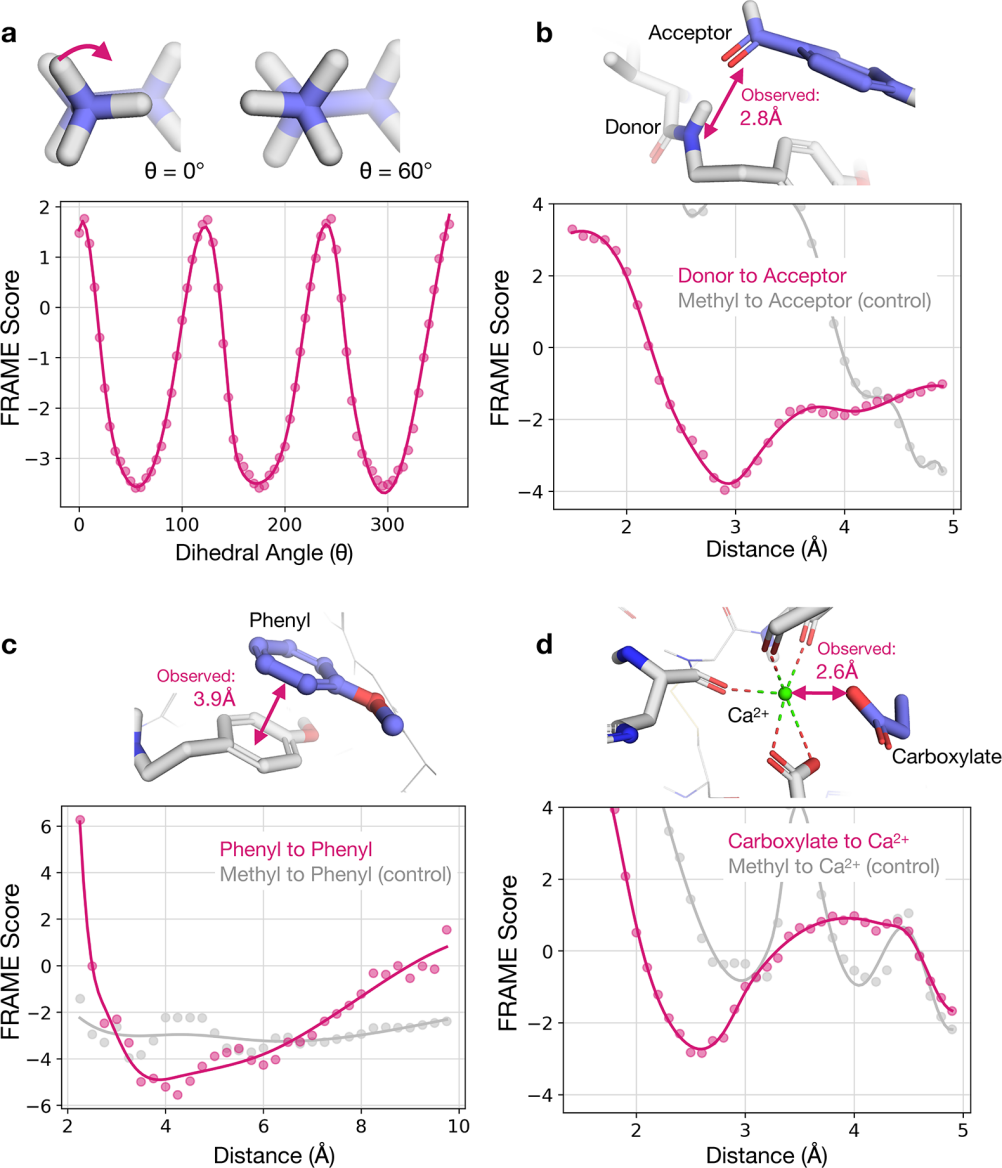
FRAME fragment scoring model learns to accurately describe specific molecular interactions despite being given no prior knowledge of such interactions. (a) The FRAME model was used to score a set of ligand-pocket structures that varied only in the dihedral angle of an attached methyl fragment. Dots represent measured scores, and the solid line is a smoothed spline curve. The lowest model scores (most favorable) correspond to the staggered conformation, which is the most energetically favorable. The highest scores correspond to the eclipsed conformation which is the least energetically favorable. (b) To test the recognition of hydrogen bonds, we varied the distance between a donor (backbone amine) and acceptor (carbonyl) atom and computed a FRAME model score for each structure. The lowest scores corresponded to reasonable distances for hydrogen bonds (approximately 3 Å). The observed distance corresponds to that in the experimental structure from which this example is derived. As a control, we replaced the acceptor (carbonyl) with a methyl group and did not observe the same behavior. (c) To test the recognition of π–π interactions, we varied the distance between the centroids of two perpendicular phenyl rings. The minimum score was close to the observed distance for π–π interactions in the experimental structure from which this example was derived (3.9 Å). As a control, we replaced one ring with a methyl group and saw no interaction. (d) To test the recognition of interactions with metal ions, we varied the distance between a carboxylate and a coordinated calcium ion. The lowest scores correspond to the metal coordination distance observed in this specific experimental structure (2.6 Å). All structures in the figure were curated from the test set.

We next analyzed the FRAME fragment scoring network, finding that it accurately describes molecular interactions (Figure 6), despite being given no prior information about such interactions or physics properties like charge. By learning these principles rather than memorizing specific atom arrangements, FRAME can generalize to unseen examples.

First, we examined whether FRAME could identify energetically favorable ligand conformations by using it to score a series of structures that varied only in the dihedral angle of a methyl group. The FRAME scores had a sinusoidal pattern that aligns with chemical intuition; the most energetically favorable conformations scored the lowest, corresponding to the staggered conformation, while the unfavorable eclipsed conformation scored the highest (Figure 6a).

FRAME also recognized intermolecular interactions, including hydrogen bonding and π–π stacking. We varied the distance between a hydrogen-bond donor on the protein and acceptor atom on the fragment by pulling the fragment away from the pocket and scored the resulting conformations (Figure 6b). The minimum FRAME score corresponded to a donor–acceptor distance of 2.9 Å, precisely within the expected range of typical hydrogen bonds. To confirm this was specific to fragments with acceptors, we repeated the test with a nonpolar methyl. This fragment had much less favorable scores and an altered minimum. Thus, the model specifically recognized the interactions between the donor and acceptor atoms, given only the chemical elements and geometries.

We performed the same type of experiment with two aromatic rings (Figure 6c). FRAME identified a ring centroid distance of about 4 Å as optimal, consistent with the geometry of typical π–π interactions in proteins. Additionally, FRAME recognizes metal–ligand interactions despite their rarity in the data set. For example, the optimal distance between a calcium ion and carboxylate group as predicted by FRAME matches the distance observed in the unseen reference structure (Figure 6d). These examples demonstrate the ability of the model to generalize and learn fundamental physical principles from small sets of molecular structures.

### Conclusions

In this work, we present a method to efficiently expand a small starting molecule bound to a protein pocket into a drug-like ligand. Our work provides a novel hypothesis generation tool for medicinal chemists to accelerate drug discovery and advances in human health.

FRAME relies on the combination of several key ideas. First, we break down the generative process into a sequence of individual actions guided by trained models. This gives our method flexibility in being applied to diverse optimization tasks whether they require adding a few final functional groups or building new scaffolds. Although molecule generation is a complex, multistep process, we train neural networks efficiently using supervised learning. Second, FRAME’s rotationally and translationally equivariant scoring networks consider 3D structure and geometry, which naturally capture the ligand within the full context of a protein pocket. Third, we train neural networks as scoring functions, which avoids encoding our fragment library within the network itself. Thus, we can easily vary the fragments for each application, and the model can be applied to new fragments.

These results come with several caveats. First, FRAME still fails to produce good ligands in some instances, resulting in worse docking scores and synthetic complexities than those of reference ligands. The autoregressive approach is not robust to occasional mistakes, such as blocking the growth trajectory or missing a critical interaction. These issues could be resolved by searching more effectively or combining our approach with more global structure generation such as denoising diffusion probabilistic models.^46^ Furthermore, many of the properties used in this work for benchmarking are themselves computational estimates or predictions, including log *P*, synthetic accessibility, and docking scores. To fully validate the effectiveness of molecules produced by FRAME and other computational methods, these molecules will ultimately need to be synthesized and their properties will need to be measured experimentally. Indeed, future work will necessitate the application of FRAME and related methods to tackle the challenges of specific, real-world drug design projects.

## Data Availability

The computational models and data sets reported in this work will be made available on GitHub.

## Supporting Information Available

Method details, Supplementary Tables 1–4, and Supplementary Figures 1–10 (PDF)
