# Supplementary material for: Geometric Deep Learning for Structure-Based Ligand Design
Source: ACS Cent Sci. 2023 Nov 17;9(12):2257–67. doi: 10.1021/acscentsci.3c00572 (PMC10755842; doi:10.1021/acscentsci.3c00572)
Supplement: Supplementary file 1 — oc3c00572_si_001.pdf [file oc3c00572_si_001.pdf]

Supporting Information for

Geometric Deep Learning for Structure-Based Ligand Design

Alexander S. Powers[1,2,3,4,5], Helen H. Yu[2,3,4,5,a,b], Patricia Suriana[2,3,4,5,a], Rohan V. Koodli[2,3,4,5,7], Tianyu Lu[6], Joseph M. Paggi[2,3,4,5], Ron O. Dror[2,3,4,5,b]

[1] Department of Chemistry, Stanford University, Stanford, CA 94305, USA
[2] Department of Computer Science, Stanford University, Stanford, CA 94305, USA
[3] Department of Molecular and Cellular Physiology, Stanford University School of Medicine, Stanford, CA 94305, USA
[4] Department of Structural Biology, Stanford University School of Medicine, Stanford, CA 94305, USA
[5] Institute for Computational and Mathematical Engineering, Stanford University, Stanford, CA 94305, USA
[6] Department of Bioengineering, Stanford University, Stanford, CA 94305, USA
[7] Biomedical Informatics Program, Stanford University School of Medicine, Stanford, CA 94305, USA

[a] H.H.Y. and P.S. contributed equally to this work
[b] To whom correspondence may be addressed. Email: ron.dror@stanford.edu

Table of Contents

Methods ***2***

Dataset Preparation 2

Fragment Library 3

Architecture 4

Training 5

Hyperparameters 5

Docking 6

Other Expansion Methods 6

Realistic Pocket Dataset 6

Supplementary Tables ***8***

Table S1. 8
Table S2. 8
Table S3. 8
Table S4. 9

Supplementary Figures ***10***

Figure S1. 10

Figure S2 ... 11
Figure S3 ... 12
Figure S4 ... 13
Figure S5 ... 14
Figure S6 ... 16
Figure S7 ... 17
Figure S8 ... 18
Figure S9 ... 20
Figure S10 ... 21

Methods

Dataset Preparation

Protein-ligand complexes were collected from PDBbind v2019. We first prepared the structures using the Schrodinger suite (v2019-2); missing sidechains were added, bond orders were determined, hydrogens were added, waters were deleted, and protonation states were determined using Epik at pH 7.0. Energy minimization was performed with non-hydrogen atoms constrained to a root mean squared deviation (RMSD) of less than 0.3 Å from the initial structure.

Ligands were then filtered to enrich for drug-like small molecules. The following criteria were used as filters: molecular weight greater than 150 Da, number of amides per molecular weight unit less than 0.0057, number of rotatable bonds less than 25. Using substructure matching with RDKit, we further removed sugars, nucleotides, lipids, and duplicates. This resulted in 4234 drug-like ligands with an average affinity of 300 nM. The ligands from this dataset are termed *reference ligands.*

We prepared the *reference ligands* for our learning framework by creating 3D *reference trajectories*. The trajectory algorithm takes as input a predetermined library of fragments (see Fragment Library) and the input molecule. First, we find all non-library fragments in the molecule. We do this by removing substructures that match library fragments as long as these substructures are not part of rings and only connected to other substructures by single bonds. Then we split apart any ring structures that are connected by single bonds. The remaining connected components of the molecular graph are considered the non-library fragments. We add these to the user library to form the full library. To generate the trajectory, we iteratively remove matched fragments from the full library that are *terminal*, they are connected to the rest of the molecule by a single bond. If there are multiple terminal fragment matches, we remove the largest one first. We continue until only a single fragment remains.

Protein pockets are prepared by (1) removing nonpolar hydrogen atoms from the reference ligands and protein (2) shifting each ligand atom by 2.5 Å in a random direction (3) selecting all protein atoms within 6 Å of any noised ligand atoms (4) extracting the full residues containing any of the selected atoms.

For the attachment location dataset, we use ligand hydrogen atoms as attachment points for new fragments. We train a classifier that predicts whether each ligand hydrogen atom should be used as an attachment point or not. Using the reference trajectories, we computed binary labels for each ligand hydrogen atom in the intermediate states, corresponding to whether this hydrogen would be replaced by a fragment in the final state (the

full reference ligand). We randomly select up to 6 intermediate states to use as examples for each trajectory.

For the fragment scoring dataset, we also randomly select up to 6 intermediate states to use as examples for each trajectory. For the positive examples (label=1), we used the reference fragments themselves or generated examples that closely matched the reference fragments by RMSD. For the negative examples (label=0), we created *decoy* examples with incorrect fragments added to the intermediate state in place of the reference fragment. Four decoys were sampled from fragments that differed chemically from the reference fragment. We also sampled 1 decoy that had the same chemical identity as the reference fragment but differed substantially in the dihedral angle (at the attachment bond) from the reference fragment. After training classifiers on the v1 dataset, we created an additional dataset for fine-tuning. We used the same positive examples as in v1. We applied the scoring network trained with v1 to rank sampled fragments; we than selected 4 decoys from the top 4 incorrect fragments. These are fragments the v1 model scores highly but that do not match the reference fragment (examples that confused the model). We added 4 additional randomly sampled decoys to round out the fine-tuning dataset. This approach was inspired by curriculum learning, which trains a machine learning model from easier data to harder data.

We computed a weighting for fragment examples using molecular interactions. We calculated the number of interactions for each example fragment, including hydrogen bonds, salt-bridges, and pi-pi interactions using the Schrodinger python API (v2019-2). We upweighted the positive examples by 1+minimum(# interactions, 3). If the reference fragment formed interactions, we weighted the decoys according to 0.25 + 1.5*(fraction interactions missing). For example, if the decoy formed the same interaction as the reference, it receives a weight of 0.25. If it makes none of the interactions, it receives a weight of 1.75. Weights are multiplied by the loss per example during training.

Feature vectors are computed for each atom in the pocket and ligand. These features include a one hot encoding of the element type, a binary flag indicating if the atom is part of the ligand or the protein, and a binary flag indicating if the atom belongs to the candidate fragment (applicable for fragment scoring model only). The one-hot encoding of the element types corresponds to the following 6 groups: C, O, N, P/S, halogens, and H. Elements not in these groups are removed. The total number of input features is 8.

Fragment Library

To obtain the fragment library, we began by defining a minimal set of functional groups, *{F}*, that are important for drug-like ligands (methyl, ethyl, phenyl, carboxy, nitro, cyclohexyl, sulfonamide etc.). We then iterate over each molecule in our dataset to find additional fragments not in *{F}*. First each molecule is converted to a graph representation. Then we remove subgraphs that match a graph in *{F}* as long as these subgraphs are not part of rings and only connected to other subgraphs by single bonds. Then we split apart any ring structures that are connected by single bonds. The remaining connected components of the molecule graph are considered the non-library fragments and added to *{F}*. We do this for each ligand in our dataset to obtain the complete library.

To use this library for expanding molecules, we also calculate each unique attachment point on the fragments. This includes chemically and geometrically distinct attachment points.

For example, axial vs. equatorial hydrogens on cyclohexane are considered as distinct attachment points. We do not yet consider multiple conformations of each fragment (chair vs. boat conformations for cyclohexane) though this can be easily incorporated in future work.

Architecture

Our architecture has two main components: (1) embedding unit and (2) aggregator unit (see Figure S9 A) inspired by Townshend *et. al.* (Science, 2021). The embedding unit consists of two layers of sequential application of self-interaction, point-convolution, point normalization, self-interaction, and nonlinearity (see Figure S9 B). Each point convolution updates the features V associated with a given point a based on the features of the 50 closest neighboring points in the Euclidian 3D space, weighted by a function of their distances and the vector between the points. The equivariant point convolution is defined as:

$$L^{l_o}_{a,c,m_0}\left(V^{l_i}_{a,c,m_i}\right) = \sum_{m_i,m_f} C^{(l_o,m_o)}_{(l_f,m_f)(l_i,m_i)} \sum_{b\,\in\, neighbors(a)} F^{l_f}_{c,m_f}(\vec{r}_{ab}) V^{l_i}_{b,c,m_i}$$

The features associated with a point have multiple components $V^{l_i}_{a,c,m_i}$ where m, c, l are indexes corresponding to angular, radial, and order features respectively. For the layers of the embedding unit, we restrict the maximum filter rotation order to $l = 2$. Features from the neighboring points, indexed by b, are weighted by an equivariant filter function and combined across multiple angular orders using Clebsch-Gordan coefficients C. The different combinations of l_f, and l_i (as controlled by the Clebsch-Gordon coefficient) can yield outputs for the same angular order l_o; the result of each combination of l_f and l_i is concatenated together along the c dimension. The filter function F combines the spherical harmonics with a radial basis function:

$$F^{l}_{c,m}(\vec{r}_{ab}) = R_c(r_{ab}) Y^{l}_{m}(\hat{r}_{ab})$$

$R_c(r_{ab})$ is a radial filter that utilizes the distance between points a and b. The radial filter uses a Gaussian radial basis function (RBF) kernel and a trainable network of two dense layers with a hidden layer (followed by a ReLU nonlinearity) of size 12. The number of basis functions and maximum radius of the Gaussian RBF kernel determines the spatial resolution of the kernel, which we chose to be 12 and 12.0 Å respectively (see Figure S9 C). $Y^{l}_{m}(\vec{r}_{ab})$ are the spherical harmonic functions where the input is the normalized vector between points a and b. Point normalization applies a normalization operation to the features of each point individually. Point nonlinearity applies a softplus and bias term to the features of each point individually. Self-interaction is also applied to the features of each point individually and mixes information across radial channels using learned weights and a bias term.

Starting from a one hot encoding the element type, ligand atom flags and candidate fragment atom flags (if applicable) as feature channels at the input, the first layer of the embedding unit mixes these features and outputs 24 feature channels per rotation sequence ($l = 0$, $l = 1$ and $l = 2$). The second layer of the embedding unit further mixes those features to output 12 feature channels per rotation order. The 0-th rotation order outputs, which

corresponds to scalar outputs, of this layer are then averaged across selected points to be passed as input to the aggregator unit.

The learned embedding features of the final layer of the embedding unit act as input to the aggregator unit. The aggregator unit consists of 2 dense layers (followed by ELU activation function except for the final dense layer) with a hidden dimension of 256 (see Figure S9 A). The aggregation process differs depending on the type of actions being evaluated. To evaluate the possibility of ligand hydrogen atoms serving as fragment attachment points, we do not perform aggregation of the learned embedding features, and the embedding features of each hydrogen atom are independently passed to the aggregator unit, which outputs a probability value for each hydrogen atom as a fragment attachment point. On the other hand, to score a candidate fragment at a given location, we first take the average of each learned embedding features over all fragment points to obtain an average embedding feature before passing it to the aggregator unit. The output of the aggregator unit is a scalar value corresponding to the score of the candidate fragment.

Training

We split the dataset into train and test sets such that no protein in the training set shares more than 30% sequence similarity with any protein in the test set. We collected sequence identity data from the Protein DataBank (PDB) using the official PDB sequence clusters (https://www.rcsb.org/docs/grouping-structures/sequence-based-clustering) determined at the 30% sequence identity cutoff with the MMseqs2 software.

Training is formulated as a binary classification task with binary cross entropy loss. To address issues with imbalanced datasets, as we have considerably more negative than positive samples, we randomly oversample the less frequent class respectively during training.

We train using the Adam optimizer in PyTorch with a learning rate of 0.001 (for attachment point selection task) and 0.01 (for candidate fragment scoring task) and a batch size of 16 for 50 epochs, and monitor the loss on the validation set at each epoch. The weights of the best-performing network (one with the lowest validation loss) are then used to evaluate the predictions on the test set. We train the models on 1 NVIDIA GeForce RTX 3090 for 4-40 hours depending on the task.

Hyperparameters

We train variants of the FRAME network where we vary the number of embedding layers in the embedding unit. The other parameters of the network architecture remain unchanged. We measure the AUPRC (Area Under Precision Recall Curve, the higher the number, the better) on the validation set and find a significant performance drop when there is only one embedding layer. FRAME with three embedding layers performs slightly better than FRAME with two embedding layers, although the gain is not significant compared to the higher computational cost (Table S4). Therefore, we chose to use two embedding layers in our final architecture.

For our fragment scoring model, we also experimented with adding a flag to indicate whether an atom is part of the candidate fragment being scored as part of the input feature. We found that adding fragment flag significantly improves the AUPRC on the test set (Table S4).

We also experimented with the aggregator function, which calculates the average of the embedding features; this averaged embedding is passed to the aggregator unit, which then calculates the final prediction (see Figure S9 A). We compared using the average over all atoms to using the average over only the candidate fragment atoms as input to the aggregator unit. We found that using the average over only the candidate fragment atoms as input to the aggregator unit achieves the best AUPRC, which makes intuitive sense since we want to predict whether the candidate fragment is suitable to be added to our ligand (Table S4).

We also experimented with the number of nearest neighbors (NN) to be included in the embedding steps; we tested NN=10,20,30, 40 and 50. We chose to use NN=50, which had the highest positive class recall.

Docking

For iterative docking and benchmarking metrics, we used Glide from Schrodinger (v2019-2) with single precision scoring (GlideScore version SP5.0) and default settings (VdW radii of ligand atoms scaled by 0.8, and charge cutoff for polarity of 0.15). Docking grids were centered at the centroid of the reference ligand with a cubic grid of side length 30 Å. For benchmarking (Figure 5a), we used the mininplace option which performs a restrained minimization prior to scoring (no pose sampling).

Other Expansion Methods

Here we discuss the details of the expansion methods. Four methods were tested. These were termed FRAME, iterative docking, random, and virtual screening. Note that for adding multiple fragments with FRAME, we use the fine-tuned version of the fragment scoring model. To increase the efficiency of all methods, we applied several fast filters. We do not add fragments if it would result in a single bond between any of the following elements: N, O, and halogens. We also remove fragment candidates if they have extreme clash with the pocket, defined as a clash volume of the fragment with the protein of greater than 15 Å^3. The random baseline method randomly samples valid fragments that pass these filters; therefore, these are not truly random ligands but a rather a naïve sampling of reasonably connected fragments that fit the pocket. For iterative docking, we used Glide from Schrodinger (v2019-2) with the settings described in the preceding section. At each step, we enumerated candidate fragment structures, performed docking (in-place mode), and selected the fragment that receives the best docking score.

For the virtual screening method, we considered all molecules from the Enamine REAL Space data set that contained the starting molecule as a substructure; these molecules could represent expansions of the starting molecule. We used the Enamine Store to check if there were any matching molecules; we selected a sample of up to 30,000 matches per example protein (which requires 4 hours per example to screen, significantly longer than FRAME). We then conducted virtual screening using Glide (HTVS precision) while restraining the matching substructures to within 1 Å of the starting molecule pose. We selected the molecule with the lowest docking score for each example protein pocket to use for benchmarking.

Realistic Pocket Dataset

We constructed an additional benchmark set of 100 proteins with structures in which the binding pocket didwere *not* already have a high-affinity, drug-like ligand bound. First, we searched the PDB to identify structural examples that might represent different stages of ligand optimization. Each such exampl required a pair of structures of the same protein, one structure with a small, fragment-like ligand bound (ligand A, structure A) and the other structure with a larger, more drug-like molecule bound (ligand B, structure B) in the same pocket. We ensured that Ligand B is always a strict expansion of ligand A. We also filtered the results to ensure that (1) ligand B was drug-like (based on the same filters described in Dataset Preparation), (2) neither ligand A nor ligand B was covalently bound to the protein, (3) ligand B contained at least 1.5x the number of heavy atoms in ligand A, and (4) none of these proteins were in the FRAME training set. Structure A thus represents what would likely be available at the beginning of a ligand optimization process, and structure B represents the end result. Examples of such structures are shown in Figure S8a, b. We then applied FRAME to generate molecules using structure A (the small ligand structure) and ligand A as a starting point. We compared the results to applying FRAME generation using structure B (the large, drug-like ligand structure) instead, again with ligand A as a starting molecule.

Supplementary Tables

Table S1. Interaction Frequency of Added Fragments by Method

Method	Hydrogen Bonds	Salt Bridges	Pi-Pi interactions
Random	0.12	0.05	0.05
FRAME	0.18	0.02	0.04
FRAME +tuning	0.4	0.06	0.07
Docking score	0.55	0.12	0.1
Reference Fragments	0.34	0.07	0.06

Value is the mean number of each type of interaction per added fragment.

Table S2. Recovery of Reference Fragment Interactions by Method

Method	Interaction Recovery Frequency (% of examples)	Extra Interaction Frequency (% of examples)
Random	15	12.81
FRAME	40.68	5.32
FRAME +tuning	78.51	12.69
Docking score	71.76	35.94

For interaction recovery, the percentage is calculated considering only examples where the reference fragment makes interactions (from the types listed in Table 1). Extra interaction frequency is calculated over all reference fragments.

Table S3. Benchmarking Results on Realistic Pockets Dataset

Property	Reference Ligands (Large ligand pocket)	FRAME Generated Ligands (Large ligand pocket)	FRAME Generated Ligands (Small ligand pocket)	Iterative docking (Small ligand pocket)
Docking score	-9.6 (0.3)	-8.2 (0.2)	-8.0 (0.2)	-10.6 (0.3)
Off-target docking score	-4.6 (0.1)	-4.9 (0.1)	-4.7 (0.1)	-4.9 (0.2)
Synthetic accessibility score	4.0 (0.1)	4.7 (0.1)	4.7 (0.1)	5.2 (0.1)
Predicted log(P)	2.6 (0.3)	0.2 (0.3)	-0.3 (0.3)	-1.35 (0.4)
Formal Charge	-0.3 (0.1)	-0.10 (0.1)	0.0 (0.1)	1.4 (0.2)
Number of Rotatable Bonds	8.1 (0.4)	8.3 (0.4)	8.3 (0.5)	7.3 (0.3)
Number of Rings	4.3 (0.2)	3.8 (0.2)	3.1 (0.2)	4.6 (0.3)
Molecular Weight	534.5 (16.5)	515.1 (18.4)	486.8 (21.5)	544.4 (19.1)

Value is the mean (s.e.m.).

Table S4. Hyperparameter Experiments

Hyperparameter	Metric
**# of Embedding Layers**	**AUPRC (on location dataset)**
1	0.88
2	0.98
3	0.99
**Fragment Flag (FF)**	**AUPRC (on fragment dataset)**
True	0.48
False	0.60
**Aggregation**	**AUPRC (on fragment dataset)**
Ligand atoms	0.60
Fragment only	0.64
**Nearest Neighbors (NN)**	**AUPRC (on fragment dataset)**
10	0.54
20	0.64
30	0.65
40	0.66
50	0.64

Validation AUPRC (Area Under Precision Recall Curve for validation dataset) results of FRAME when trained with different hyperparameters. For AUPRC, a higher value indicates better performance.

Supplementary Figures

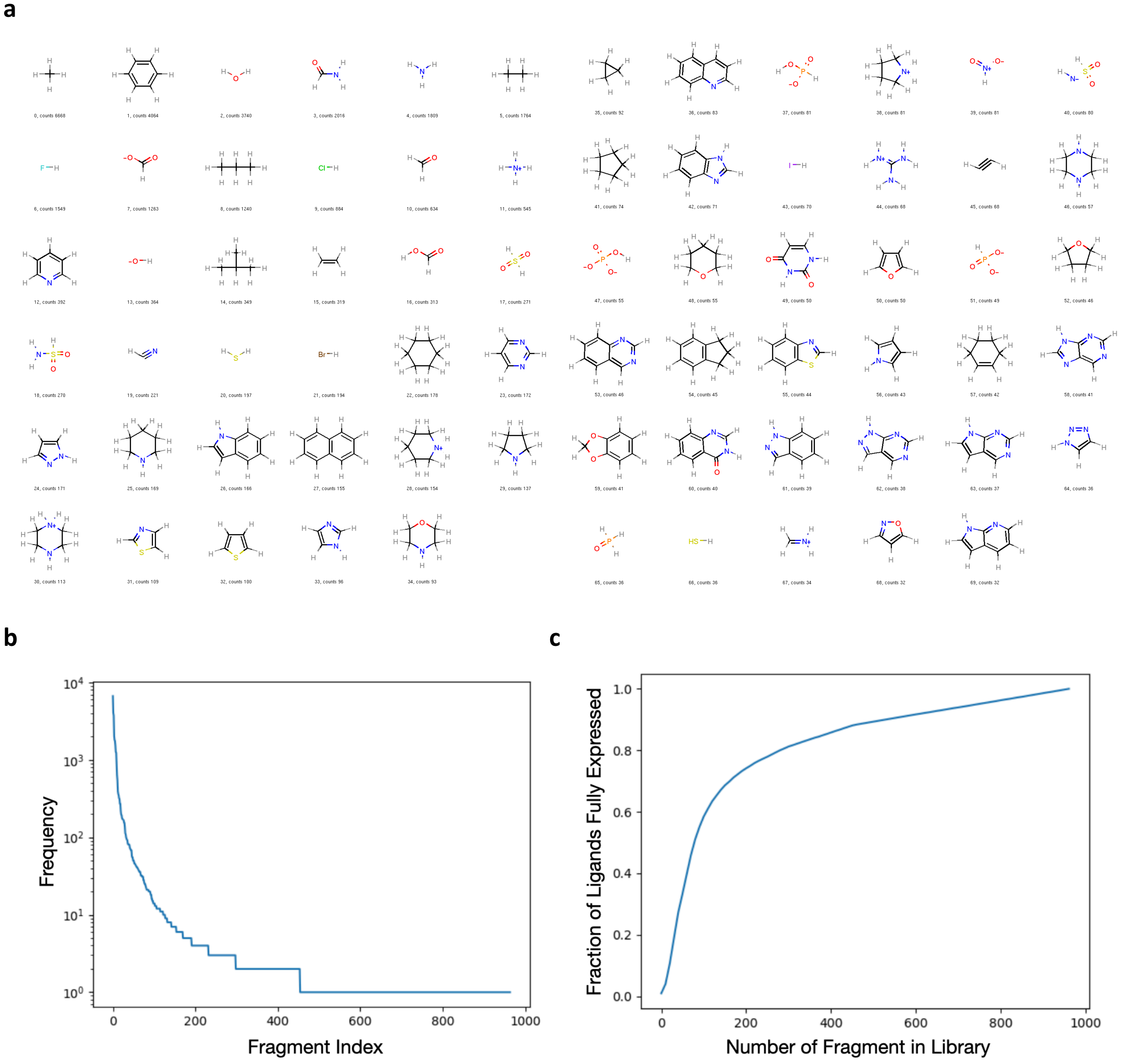

Figure S1. Many drug-like ligands are made of up of similar fragments. (a) 70 most frequent fragments in the library obtained from custom fragmentation algorithm applied to drug-like ligands. The label below each image indicates the number of occurrences of the fragments in the dataset. **(b)** Frequency distribution of all fragments in the dataset. Frequency corresponds to number of occurrences. **(c)** This plot shows how many ligands in the dataset can be fully reconstructed with a fragment library of a particular size. A majority of molecules in the dataset can be fully reconstructed by a library of less than 100 fragments.

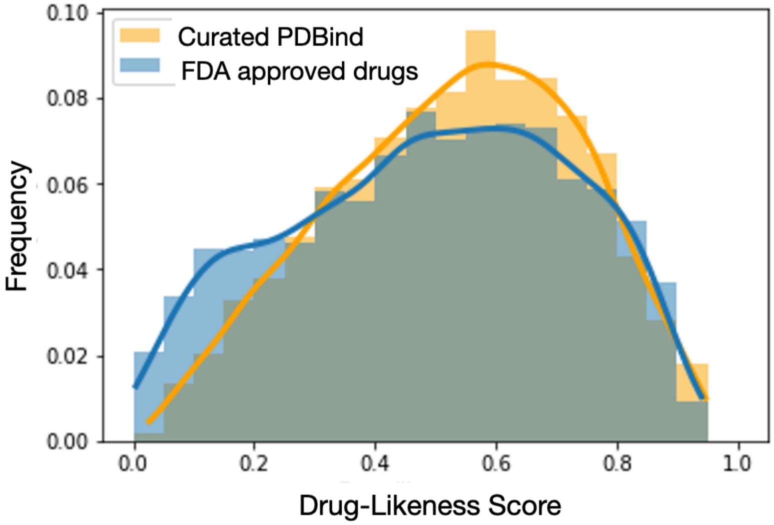

Figure S2. Curated dataset of ligands matches the properties of known drugs. We calculated Drug-Likeness Scores using RDKit (Quantitative Estimate of Drug-likeness, QED) for a list of FDA approved small-molecule drugs and for the ligand in our filtered dataset. Results are presented as as overlaid normalized histograms (colored transparent bars) with smoothed kernel density estimates of the probability distribution (solid curves).

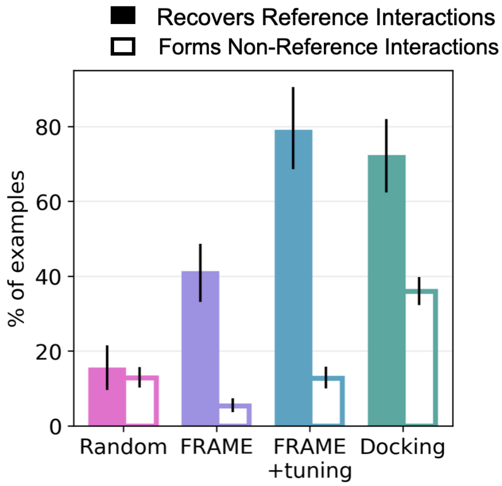

Figure S3. Recovery of Reference Fragment Interactions by FRAME. We evaluated the fragment-scoring model by measuring how often the top-ranking fragment recovered the interactions in the reference ligand fragment (filled bars). We also assessed if the top-ranking fragment formed extra interactions not present in the reference fragment (outlined bars). We compared FRAME to a naïve baseline (Random), a version of the model fine-tuned with weighted examples (FRAME +tuning), and docking scores (Docking). The analysis used 100 ligand-protein complexes from the test set, and error bars indicate the 95% confidence interval obtained from bootstrapping.

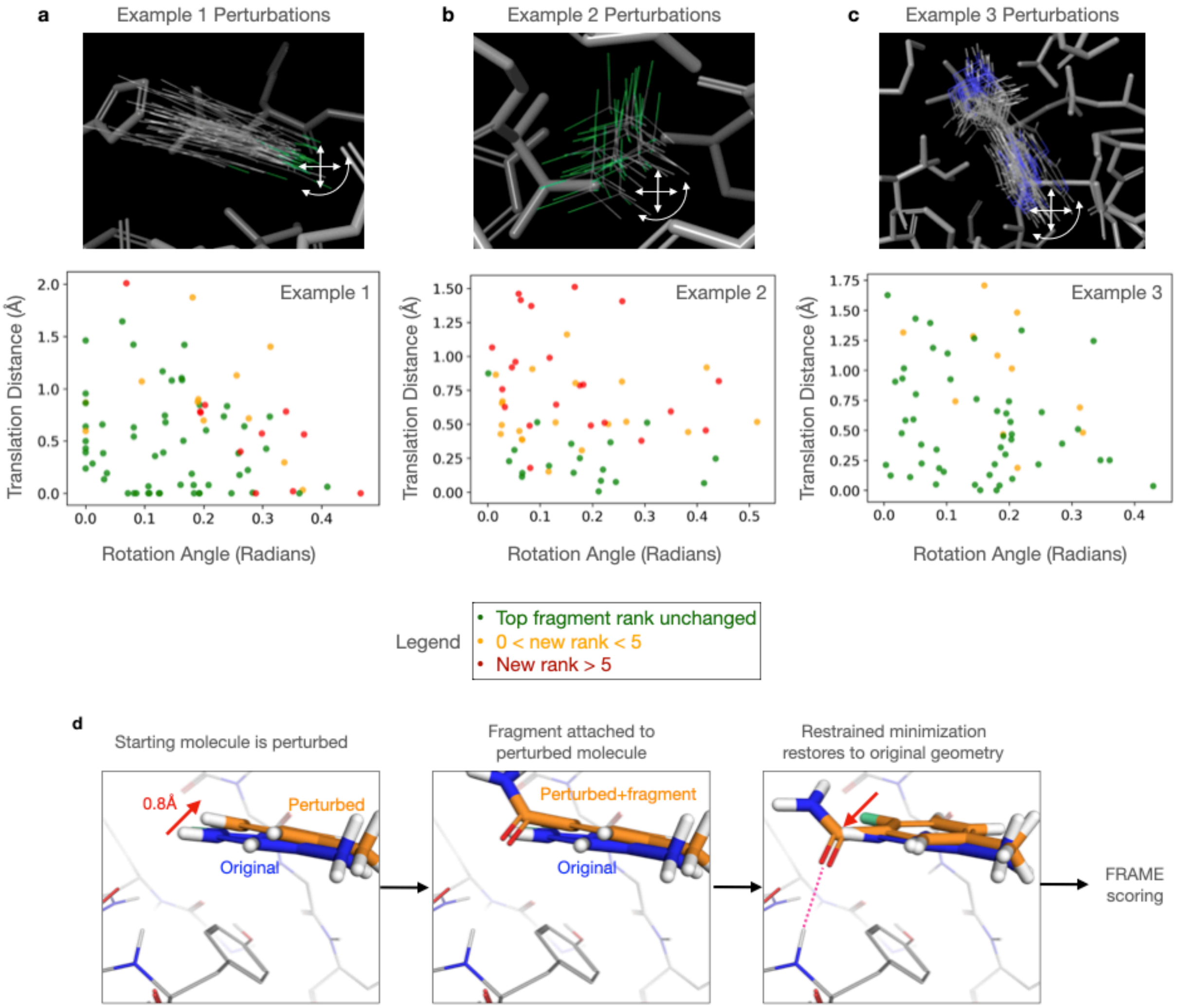

Figure S4. FRAME fragment scoring model is robust to small perturbations of the input fragment. (a,b,c) For three example input fragments, we applied random translations and rotations; each point in the scatter plots corresponds to a particular perturbation for that example. Translation distance is the magnitude of the translation vector applied to the fragment centroid. Rotation angle is the magnitude of the angle the fragment is rotated around the rotation axis (which is also randomly selected from a uniform distribution on the unit sphere). We then applied the FRAME scoring model for each new input structure to rank the next fragments to add. We observed whether the top-ranking fragment was unchanged (green dots), had a small change in rank (moved to a rank between 0 and 5, yellow dots), or a large change in rank (rank greater than 5, red dots). **(d)** To better handle significant perturbations to the starting molecule's location, we discovered that it is effective to first carry out a restrained force-field minimization of the ligand coordinates before FRAME scoring. In the example shown, FRAME selects an amide fragment for the original, unperturbed starting molecule. However, if the starting molecule is rotated from its original position significantly, the newly added amide may not create a hydrogen bond with the pocket, which can cause confusion for FRAME. By executing a minimization process, the molecule's structure is brought closer to its original location, enabling the hydrogen bond to form, and ensuring accurate FRAME scoring.

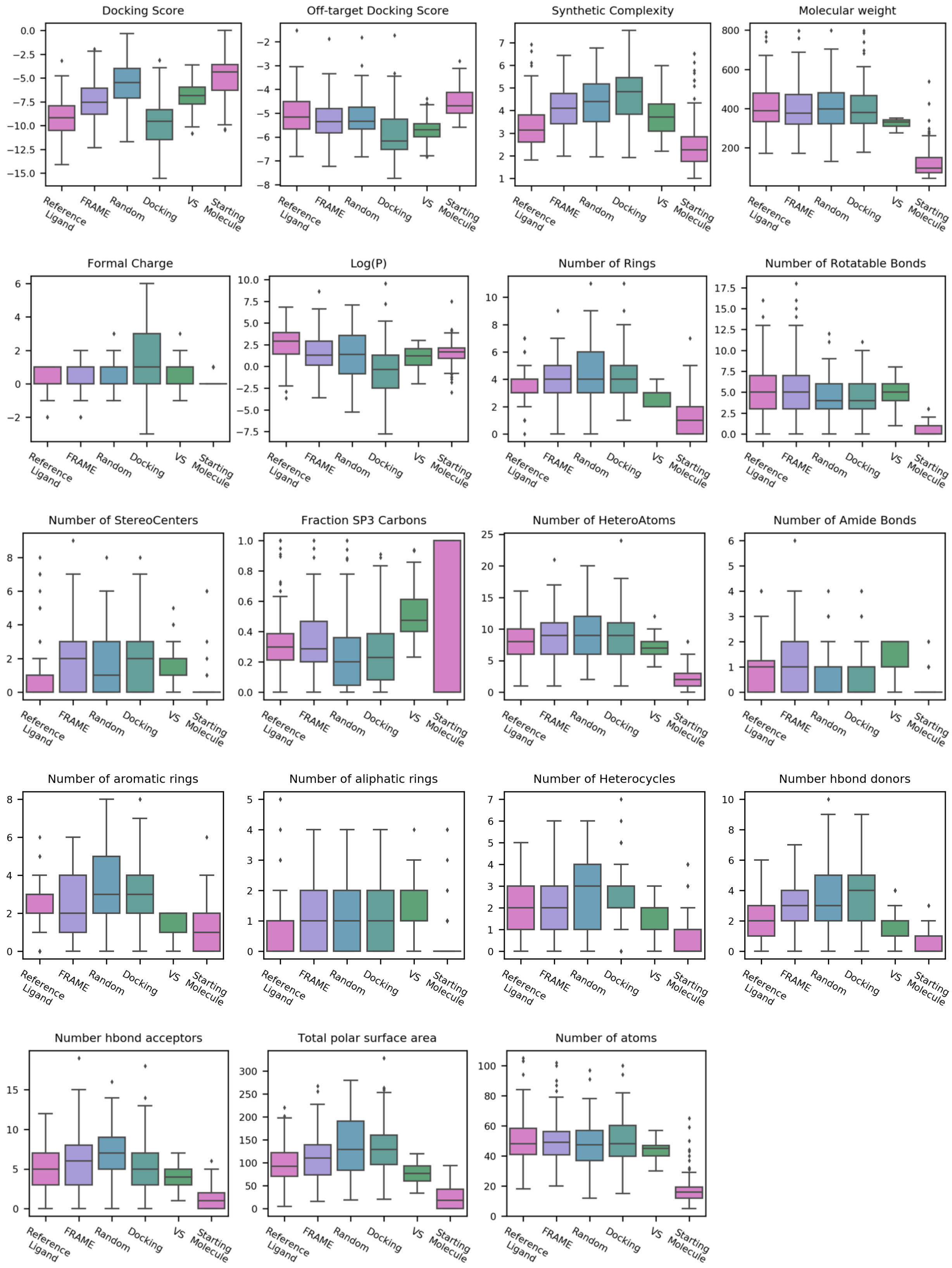

Figure S5. Ligands generated with FRAME are similar to real drug-like ligands across many chemical properties. 100 ligands were generated using FRAME, using 100 unique fragment-pocket structures

from the test set. The property distributions of the generated ligands were compared to those of ligands generated with a naive probabilistic fragment selection function (Random), iterative docking (Docking), virtual screening of the Enamine REAL space (VS), the starting molecules prior to expansion (Starting Molecule), and reference ligands from the test set (Reference Ligand). Box and whisker plots show the property distribution of the 100 generated ligands for each method. The middle line of each colored box in the plot is the median RMSD, with the box extending from the 1st to the 3rd quartile and defining the "interquartile range." Whiskers extend to last data points that are within 150% of the interquartile range, and outlier data points beyond those are shown individually. Docking scores were computed with Schrodinger's Glide software. All other properties were computed using the standard implementation in the RDKit Python library.

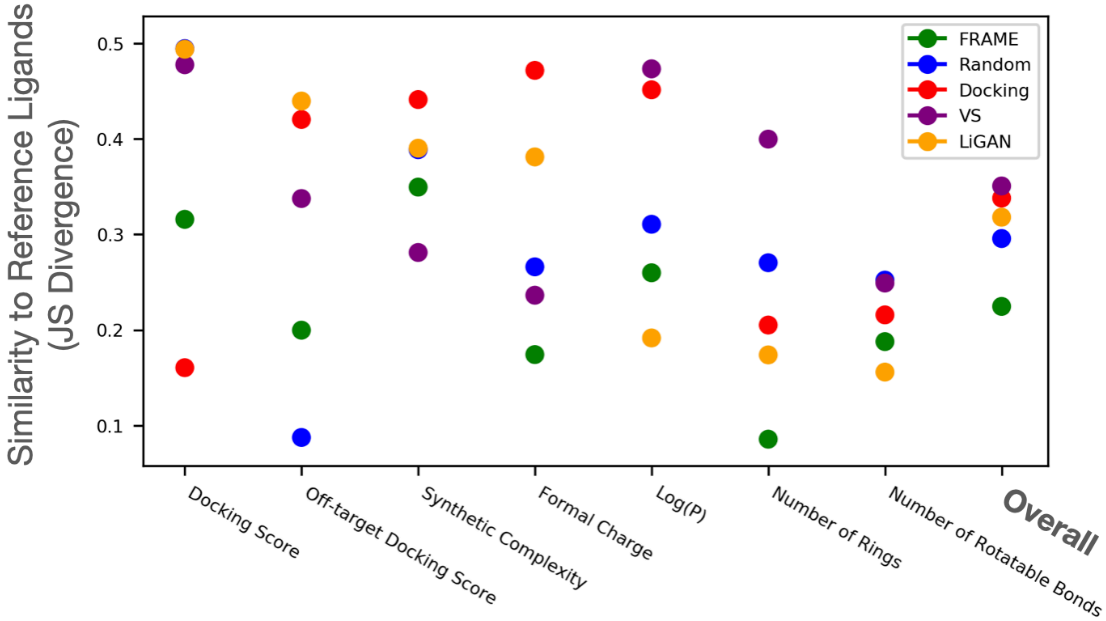

Figure S6. Ligands expanded by FRAME have the highest overall quality relative to other expansion methods. 100 ligands were expanded using each method. For each property and method, we computed the similarity of the property distribution to the reference ligand distribution using the Jensen–Shannon divergence (a bounded measure of the similarity of two distributions). Lower values reflect higher similarity. The overall similarity was calculated by averaging the scores for the 8 properties. Methods include FRAME, iterative docking (Docking), a naive probabilistic fragment selection function (Random), virtual screening (VS), and an alternative machine learning method that uses density grids (LiGAN). FRAME had the highest overall similarity to reference ligands compared to the other methods evaluated.

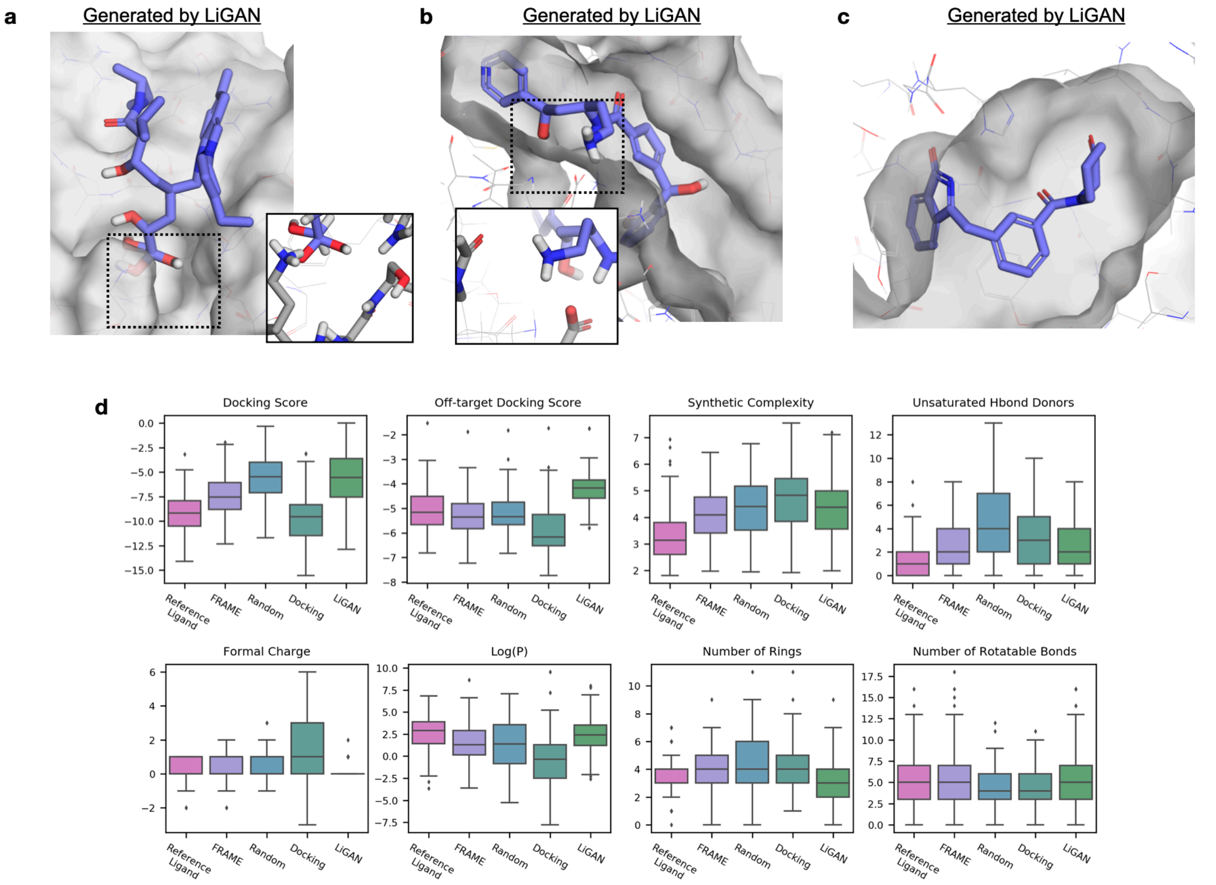

Figure S7. Evaluation of ligands produced using LiGAN. LiGAN was used to generate ligands given the protein pocket. Examples of the resulting molecules are shown in the middle column (See Figure 4 for corresponding molecules produced by FRAME). (a) Protein is HCV Protease (PDB: 3P80). (b) Protein is Protein Kinase A (PDB: 1X4H) (c) Protein is PAR Polymerase 1 (PDB: 3C49) (d**)** 100 ligands were generated using LiGAN, using 100 unique pocket structures from the FRAME test set (this gives an advantage to LiGAN as some of these proteins are in the LiGAN training set). The property distributions of the generated ligands were compared to those of ligands generated with FRAME, iterative docking (Docking), a naive probabilistic fragment selection function (Random), and reference ligands from the test set. Properties correspond to those described in Figure 5.

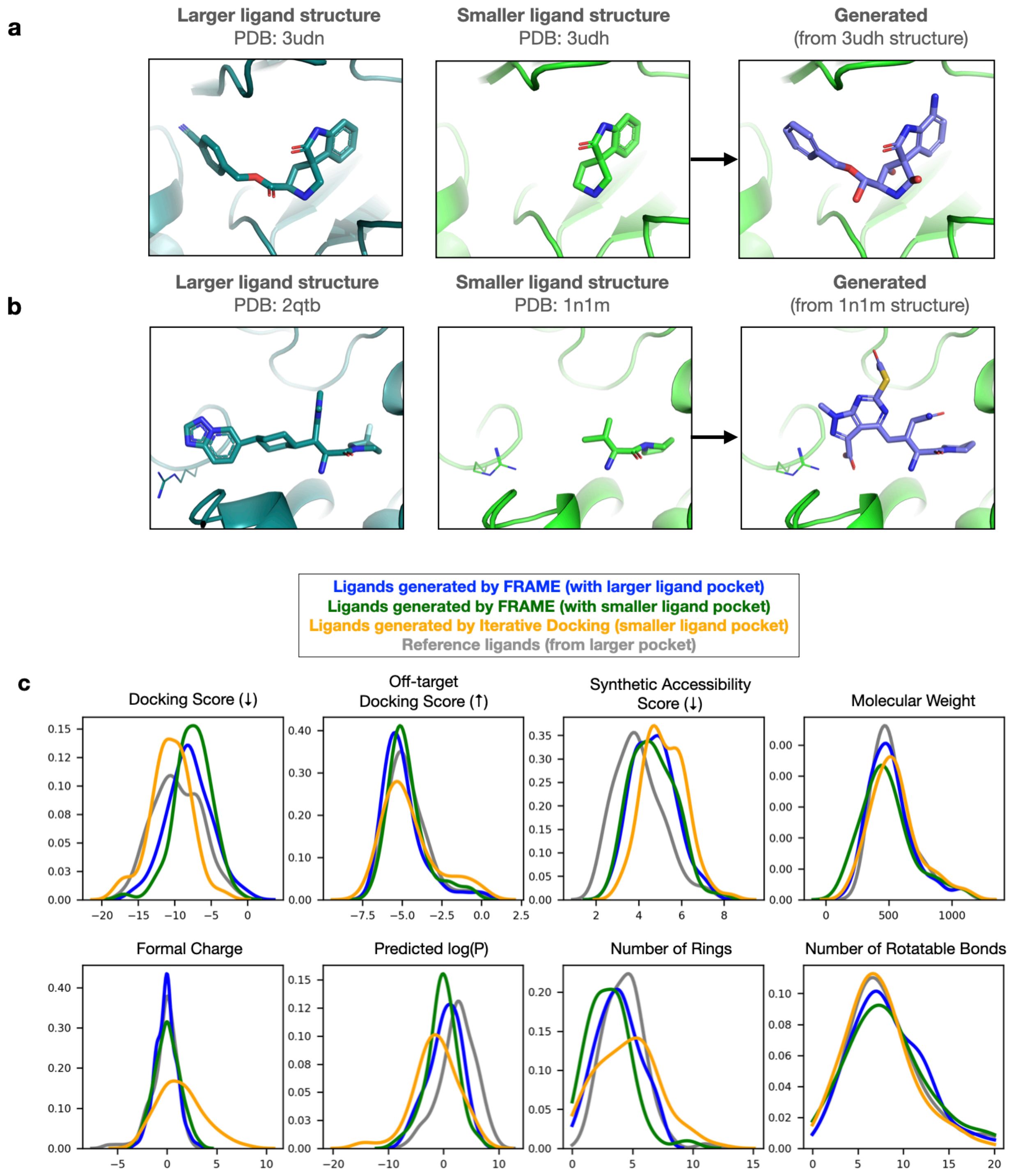

Figure S8. FRAME performance does not depend on the ligand used to determine the protein structure. (a, b) We mined the PDB to curate structural examples from different stages of ligand optimization that were not part of the FRAME training set. These examples required a pair of structures, one with a small fragment-like ligand bound (smaller ligand, middle column) and a separate structure with a larger more drug-like molecule bound (larger ligand, left column) where both ligands bind to the same protein and pocket. We ensured that the smaller ligand is always an expansion of the larger ligand. The smaller ligand

structure thus represents what would likely available at the beginning of a drug optimization process, and the larger ligand structure represents the end result. Two examples from this dataset are shown. We then applied FRAME to generate molecules using the small ligand and its structure as a starting point (shown in the right column). **(c)** Using benchmarking metrics described in the main text, we compared the results to using iterative docking on the smaller ligand structure. We also compared to applying FRAME generation using the larger ligand structure. There was not a significant difference in the results obtained from each structure across our benchmark metrics. Iterative docking again produced ligands that were polar, charged, and complex than FRAME ligands or reference ligands.

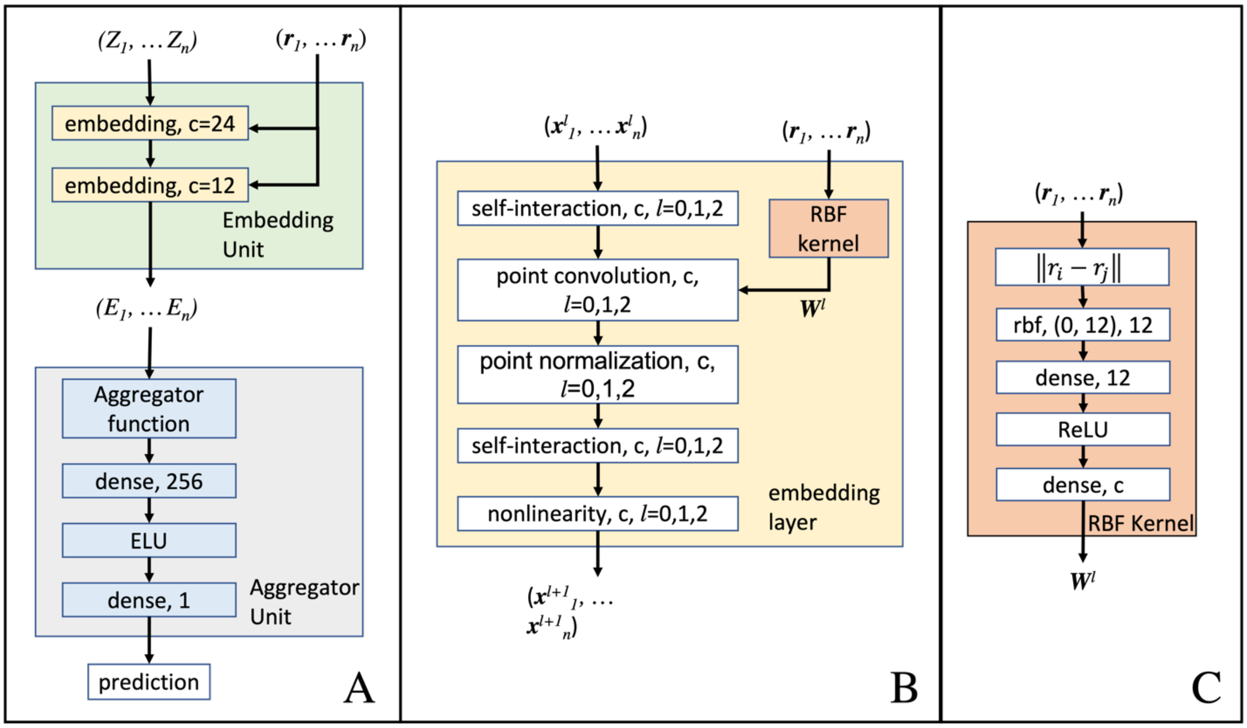

Figure S9. Architecture of the FRAME network. Panel A shows the overall architecture of FRAME, which consists of (1) an embedding unit and (2) an aggregator unit. We represent a protein-ligand complex as a set of points in 3D with associated scalar features (Z_1, ..., Z_N). The embedding unit learns an embedding features for each point through two embedding layers (panel B), each with output channels of size 24 and 12 respectively. The outputs of the embedding unit are passed to the aggregator unit to calculate the final prediction. The aggregator function of this unit depends on the task. Panel B shows the architecture of the embedding layer. The embedding layer updates the features associated with each point with respect to the features of the 50 nearest neighbor points in 3D Euclidean space, weighted by the distances of the neighbors to the point. Panel C shows the Gaussian radial basis function (RBF) kernel, a trainable network consisting of a hidden dense layer of size 12, a ReLU nonlinearity, and a final dense layer. This kernel computes the weights based on the distances of each point for updating the point convolution (panel B). The number of basis and maximum radius of the Gaussian RBF kernel determines the spatial resolution of the kernel, which we chose to be 12 and 12.0 Å respectively.

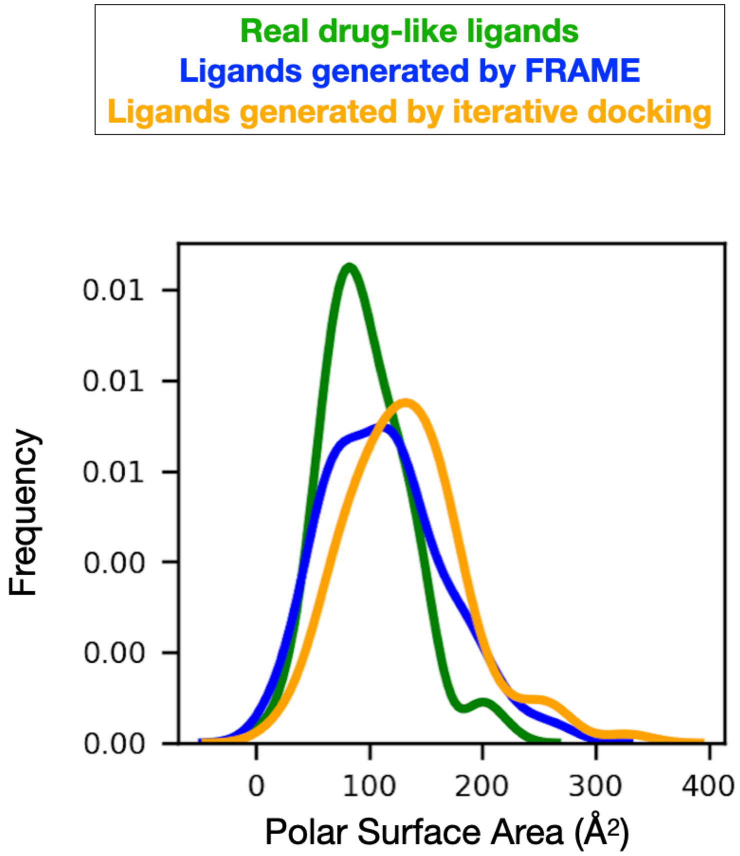

Figure S10. Polar surface area of generated ligands. 100 ligands were expanded using FRAME, using 100 unique structures from the test set. The total polar surface area distributions of the expanded ligands were compared to those of ligands expanded with iterative docking. The property distributions of the reference ligands are also included (Real drug-like ligands). Polar surface area was computed with RDKit.
